# Health and economic impact of HPV 16 and 18 vaccination and cervical cancer screening in India

**DOI:** 10.1038/sj.bjc.6604462

**Published:** 2008-07-08

**Authors:** M Diaz, J J Kim, G Albero, S de Sanjosé, G Clifford, F X Bosch, S J Goldie

**Affiliations:** 1 Unit of Infections and Cancer (UNIC), Cancer Epidemiology Research Programme, Catalan Institute of Oncology (ICO), Av. Gran Via, s/n km. 2.7, 08907 L'Hospitalet de Llobregat, Barcelona, Spain; 2 Department of Health Policy and Management, Program in Health Decision Science, Harvard School of Public Health, 718 Huntington Avenue, 2nd Floor; Boston, MA 02115, USA; 3 Department of Paediatrics, Obstetrics, Gynaecology and Preventive Medicine, Program in Public Health and the Methodology of Biomedical Research, Universitat Autónoma de Barcelona (UAB), Bellaterra 08193 (Cerdanyola del Vallès), Spain; 4 IDIBELL, CIBERESP, Barcelona, Spain; 5 Infections and Cancer Epidemiology Group, Epidemiology and Biology Cluster, International Agency for Research on Cancer (IARC), 150 Cours Albert Thomas, Lyon CEDEX 08 69372, France

**Keywords:** HPV, cost-effectiveness, vaccination

## Abstract

Cervical cancer is a leading cause of cancer death among women in low-income countries, with ∼25% of cases worldwide occurring in India. We estimated the potential health and economic impact of different cervical cancer prevention strategies. After empirically calibrating a cervical cancer model to country-specific epidemiologic data, we projected cancer incidence, life expectancy, and lifetime costs (I$2005), and calculated incremental cost-effectiveness ratios (I$/YLS) for the following strategies: pre-adolescent vaccination of girls before age 12, screening of women over age 30, and combined vaccination and screening. Screening differed by test (cytology, visual inspection, HPV DNA testing), number of clinical visits (1, 2 or 3), frequency (1 × , 2 × , 3 × per lifetime), and age range (35–45). Vaccine efficacy, coverage, and costs were varied in sensitivity analyses. Assuming 70% coverage, mean reduction in lifetime cancer risk was 44% (range, 28–57%) with HPV 16,18 vaccination alone, and 21–33% with screening three times per lifetime. Combining vaccination and screening three times per lifetime provided a mean reduction of 56% (vaccination plus 3-visit conventional cytology) to 63% (vaccination plus 2-visit HPV DNA testing). At a cost per vaccinated girl of I$10 (per dose cost of $2), pre-adolescent vaccination followed by screening three times per lifetime using either VIA or HPV DNA testing, would be considered cost-effective using the country's per capita gross domestic product (I$3452) as a threshold. In India, if high coverage of pre-adolescent girls with a low-cost HPV vaccine that provides long-term protection is achievable, vaccination followed by screening three times per lifetime is expected to reduce cancer deaths by half, and be cost-effective.

More than 25% of cervical cancer cases worldwide occur in India (Ferlay *et al*, 2004). Although cervical cancer is the most frequent cancer diagnosis in Indian women, age-adjusted incidence rates vary between regions, ranging from 10.9 in Trivandrum to 47.2 per 100 000 person-years at risk in Chennai (Parkin *et al*, 2005).

Persistent infection with high risk types of human papillomavirus (HPV) is established as the key causal agent for cervical cancer (IARC, 2005). In India, overall HPV prevalence in women with normal cytology has been estimated to be 7.5% (de Sanjosé *et al*, 2007). Similar to cancer incidence, region-specific prevalence of HPV varies considerably (Sankaranarayanan *et al*, 2004; Franceschi *et al*, 2005; Laikangbam *et al*, 2007), likely attributable to genetic and cultural diversity, as well as heterogeneity between studies (Basu *et al*, 2003; Duttagupta *et al*, 2004).

New options for cervical cancer prevention motivate important questions in low-income countries. Traditional cytology screening, conducted at frequent intervals and requiring multiple clinic visits for women with abnormal cytology (e.g., screening, diagnostic testing, and treatment), has been difficult to implement in India (Sankaranarayanan *et al*, 2001). Screening with HPV DNA testing and/or visual inspection with acetic acid (VIA) have been demonstrated to be acceptable and promising alternatives when embedded in a screening protocol that requires fewer visits (Bhatla *et al*, 2007; Ramachandran *et al*, 2007) and utilises cryotherapy conducted by nurses to treat the majority of early cancer precursors (Sankaranarayanan *et al*, 2001, 2006, 2007a, 2007b). These strategies, less dependent on existing health system infrastructure and associated with greater rates of follow-up, are also expected to be cost-effective (Goldie *et al*, 2005).

Most recently, two new vaccines have been shown to be highly effective in preventing infection with HPV 16 and 18 in women without prior exposure to these types (FDA, 2006). Contemplating an HPV vaccination programme adds complexity to decision-making about India's national approach to cervical cancer control. The country will want to consider the burden of cervical cancer, the comparative effectiveness – and potential synergies – of vaccination and screening, the financial costs required to initiate and sustain programs, their cost-effectiveness, and the programmatic capacity and infrastructure necessary to effectively deliver a three-dose pre-adolescent vaccine. Although a single study would be unable to include all of these factors, decision-analytic models can be used to synthesise the best available epidemiologic, clinical and economic data, and project long-term health and economic outcomes expected with different cancer prevention strategies. To provide insight to decision makers and stakeholders invested in reducing mortality from cervical cancer in India, we assessed the potential avertable burden of disease and cost-effectiveness associated with various vaccination and screening strategies.

## Materials and methods

### Analytic overview

A previously described computer-based model of cervical carcinogenesis (Goldie *et al*, 2007; Kim *et al*, 2007a) was calibrated to epidemiologic data in India and used to compare the health (cancer incidence reduction, life expectancy gains) and economic (discounted lifetime costs) impact of the following strategies: (1) HPV 16,18 vaccination of girls below age 12 (herein referred to as pre-adolescent vaccination), (2) screening of adult women over age 30 using HPV DNA testing, cervical cytology, or VIA, and (3) combined vaccination and screening. We followed published recommendations for economic evaluations (DCPP; Gold *et al*, 1996; Drummond *et al*, 2005; WHO CHOICE) by adopting a societal perspective and discounting future costs and life years by 3% annually. Performance of alternative strategies was measured using incremental cost-effectiveness ratios, defined as the additional cost of a specific strategy (in 2005 international dollars, I$), divided by its additional benefit (per woman life expectancy gain), compared with the next most costly strategy. Parameter uncertainty was evaluated using one- and two-way sensitivity analysis, and probabilistic analysis.

### Model

The individual-based stochastic model has been previously described (Goldie *et al*, 2007; Kim *et al*, 2007a), with the natural history of cervical cancer represented as a sequence of monthly transitions between mutually exclusive health states. Individual girls representative of a single birth cohort enter the model at age 9, before sexual debut, and are followed throughout their lifetimes. Transitions between health states depend on HPV type, age, and history of prior type-specific infection (i.e., natural immunity). Human papillomavirus types were categorized as follows: (1) high-risk type 16 (HR 16); (2) high-risk type 18 (HR 18); (3) other high-risk types (HR other) (HR types: 26, 31, 33, 35, 39, 45, 51, 52, 53, 56, 58, 59, 66, 68, 73, and 82 (IS39 and MM4 subtypes)), and (4) low-risk types (LR) (LR types: 6, 11, 30, 32, 34, 40, 42, 43, 44, 54, 55, 57, 61, 64, 67, 69, 70, 71 (CP8061), 72, 81 (CP8304), 83 (MM7), and 84 (MM8), cand85, 86, cand89 (CP6108) and JC9710) (Muñoz *et al*, 2003). Women with infection with high-risk types and high-grade cervical disease may progress to invasive cancer, and may then be detected through symptoms or screening, be diagnosed and treated, or progress to the next stage of cancer. Women with cancer face stage-specific survival rates, although all women face all-cause age-specific competing mortality risks.

### Epidemiological data

We assumed that the mechanism of cervical carcinogenesis does not fundamentally differ between countries; as such, initial natural history model estimates, and their plausible ranges, were based on the best available data regardless of specific setting. However, as epidemiology (e.g., HPV type distribution), risk factors (e.g., age at sexual debut, number of sexual partners), and age-specific cervical cancer rates differ between regions, we then calibrated the model to country-specific data. We capitalised on the availability of data from southeastern India for nearly all epidemiological targets required for our calibration procedure. Age-specific and type-specific prevalence of HPV in women with normal cytology and age-specific prevalence and type distribution of cervical intraepithelial neoplastia (CIN) lesions were derived from the IARC survey in the Tamil Nadu state (Franceschi *et al*, 2005); HPV type distribution in cervical cancer was extracted from a case–control study in the same state (Franceschi *et al*, 2003). Cancer incidence data were drawn from two population-based cancer registries from southern India, Bangalore (Karnataka) and Chennai (Tamil Nadu), reported in the Cancer Incidence in Five Continents (CI5C) (Parkin *et al*, 2005).

**Figure 1:**
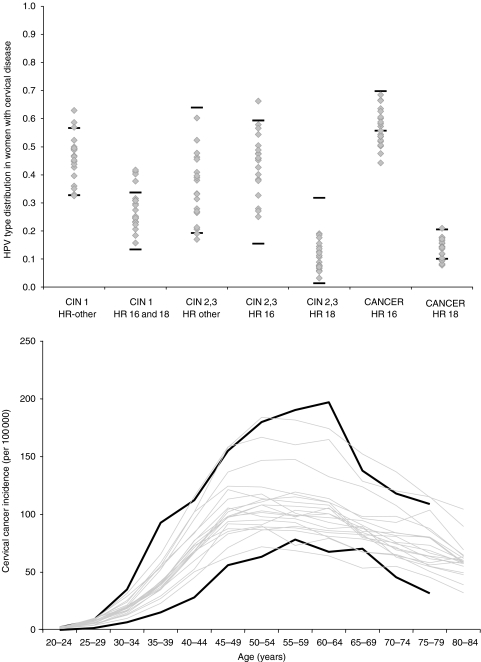
Model calibration. Selected model output from a random sample of good-fitting parameter sets are compared with the 95% confidence intervals of the empirical data (solid black lines) including HPV type distribution in cervical disease (upper panel) and age-specific cancer incidence rates (lower panel). Additional calibration results can be found in the Supplementary Appendix.

Details of the model parameterisation process can be found in the Supplementary Appendix and previous publications (Goldhaber-Fiebert *et al*, 2007; Goldie *et al*, 2007; Kim *et al*, 2007a). Briefly, after initial values and plausible ranges for each model input parameter were established, repeated simulations were undertaken, each drawing different combinations of parameter values and projecting model outcomes with each set of parameter combinations. The outcomes produced by each parameter set were scored according to their fit with multiple calibration target data based on likelihood scoring functions. A composite goodness-of-fit score for each parameter set was computed by summing the log likelihood of each model outcome. Figure 1 shows examples of model output from a sample of a good-fitting parameter sets compared with the empirical data. Additional results may be found in the Supplementary Appendix.

To explicitly incorporate the effect of parameter uncertainty, cost-effectiveness analyses were conducted with a random sample of good-fitting parameter sets, and results were reported as the mean and range of outcomes, whereas incremental cost-effectiveness ratios were reported as the ratio of the mean costs divided by the mean effects of one strategy *vs* another across the good-fitting parameter sets (Stinnett and Paltiel, 1997).

### Strategies

Vaccination occurs before sexual debut (before age 12) and may or may not be combined with screening. In the base case analysis, we elected to assume 70% of the birth cohort was successfully vaccinated with three doses; in doing so, we aimed to (i) establish an estimate for the avertable burden of disease with optimal delivery and implementation of the intervention without making assumptions about the differential operational capacity to deliver the vaccine, and (ii) allow for comparison with other cost-effectiveness analyses in the literature. We also assumed vaccinated girls have life-long protection against HPV 16 and 18, but are subject to the same rate of infection with other high-risk HPV types as those who are not vaccinated. We explored the implications of waning immunity and different levels of coverage (10–90%) and vaccine efficacy (50–100%).

Screening strategies differ by the initial screening test (cervical cytology, VIA, HPV DNA testing), screening frequency (once, twice, three times per lifetime), target ages (35, 40, and 45), and the number of clinical visits (1, 2 or 3) required for women to be screened, be informed of results, and receive any necessary treatment. In the base case, we assumed 70% screening coverage starting at age 35 with subsequent screens occurring at 5-year intervals. In the three-visit cytology strategy, women are screened in the first visit, and those who are screen-positive undergo colposcopy/biopsy in a second visit, followed by treatment of abnormalities at a district or tertiary clinical care site in a third visit. Treatment for precancerous lesions or cancer depends on lesion size and type (e.g., cryosurgery, loop electrosurgical excision procedure, cold knife conisation, or simple hysterectomy). In the two-visit HPV DNA testing strategy, women are screened in the first visit, return for results in a second visit, and screen-positive women who are eligible for cryosurgery are treated on the same day; those who are not (e.g., lesions covering over 75% of the cervix or extending to the vaginal wall) are referred to a secondary facility for further diagnostic testing and, potentially, treatment. Loss to follow-up between each visit is assumed to be 15%. One-visit strategies (VIA and rapid HPV DNA testing) incorporate same-day screening and treatment for women with positive screening results.

### Cost data

Selected cost estimates were based on data from a previously published analysis of screening in India (Goldie *et al*, 2005) (Table 1 and Supplementary Appendix). Costs from Goldie *et al* (2005), which were in I$2000 international dollars (I$), were converted to I$2005 using Purchasing Power Parity (PPP) conversion rates and the gross domestic product (GDP) implicit price deflator (World Bank). Since the HPV vaccine price and the programmatic costs to deliver a pre-adolescent vaccine in India are not yet known, we expressed a composite value, the ‘cost per vaccinated girl’, and varied it from I$5 to I$360. For example, for a composite cost of I$10 per vaccinated girl, we assumed three doses of vaccine at $2.00 each; wastage of $0.90; freight and supplies of $0.59; administration of I$0.50; and immunisation support and programmatic costs of I$2.00. All costs were expressed in I$2005.

## Results

### Reduction in lifetime risk of cancer

**Figure 2:**
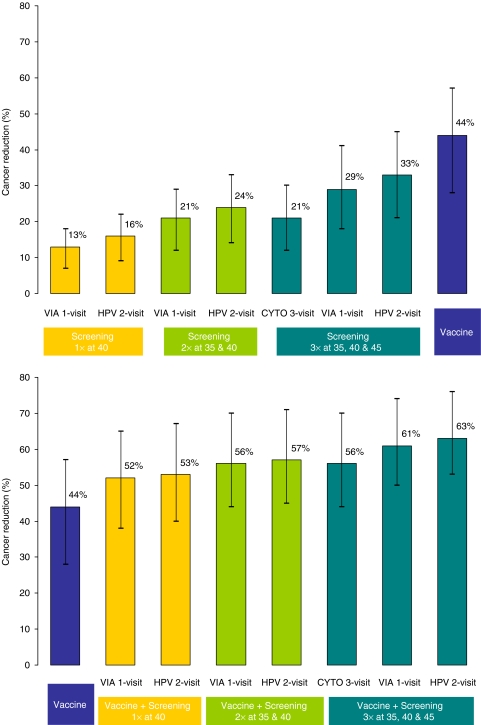
Reduction in lifetime risk of cervical cancer. The mean reduction in lifetime risk of cervical cancer is shown with strategies using either vaccination or screening (upper panel), and strategies combining both vaccination and screening (lower panel). The range represents the minimum and maximum reductions achieved for each strategy across the good-fitting parameter sets.

Pre-adolescent vaccination alone reduced cancer incidence by 44% (range, 28–57%) and was more effective than screening alone (Figure 2, upper panel). A combined approach of pre-adolescent vaccination and screening of adult women was more effective than either alone (Figure 2, lower panel). The relative differences between individual testing strategies were attenuated in the presence of widespread vaccination.

### Cost-effectiveness of vaccination and screening

Table 2 displays the cost-effectiveness results (cost per year of life saved (YLS)), as the cost per vaccinated girl is varied. In addition to conducting analyses that assumed all screening tests would be equally available (Table 2a), we performed analyses that assumed only HPV DNA testing was available (Table 2b). Although there is no consensus on a specific cost-effectiveness threshold, below which an intervention would be considered cost-effective, one suggested heuristic has been to use the country's per capita GDP (WHO, 2001). Although realistically the threshold ratio may need to be much lower for the intervention to be affordable, we considered strategies that had cost-effectiveness ratios lower than the per capita GDP in India (I$3452) (World Bank) to be cost-effective.

#### All strategies equally available

Assuming that all strategies were equally available (Table 2a) and provided the cost per vaccinated girl was I$10 ($2 per dose) or less, vaccination alone was more effective and cost-effective than screening alone. Vaccination and screening three times per lifetime with VIA was I$290 per YLS. When the cost per vaccinated girl reached and exceeded I$20, screening three times per lifetime (at ages 35, 40, and 45) with a single-visit VIA strategy was less costly and more cost-effective than vaccination alone, and cost I$60 per YLS compared to no intervention. At a vaccine price per dose of approximately $100 (I$360 per vaccinated girl), vaccination was dominated by (i.e., either less costly and less cost-effective than or more costly and less effective than) screening alone. The cost-effectiveness ratio for a combined vaccination and screening strategy with single-visit VIA increased from I$290 per YLS at I$10 per vaccinated girl to I$7230 per YLS at I$360 per vaccinated girl.

#### Only HPV DNA testing available

In some settings, reliable VIA screening and treatment in a single visit may not be feasible. If only HPV DNA testing was available (Table 2b), provided the cost per vaccinated girl was below I$50, screening alone was dominated. Vaccination alone ranged from cost saving at I$10 per vaccinated girl ($2 per dose) to I$890 per YLS at I$50 per vaccinated girl. Across this same range of vaccine costs, pre-adolescent vaccination combined with two-visit HPV DNA testing three times per lifetime was I$1780 per YLS; at higher costs (I$360 per vaccinated girl) this strategy increased to I$7650 per YLS, compared to the next best strategy.

Once the cost per vaccinated girl reached I$50, screening alone was no longer dominated. In this case, screening three times per lifetime with two-visit HPV DNA testing was I$720 per YLS, compared to no intervention. At a cost per vaccinated girl of I$75 and above, vaccination alone was no longer cost-effective.

### Sensitivity analysis

Assuming a cost of I$20 per vaccinated girl, if VIA screening was 30% less sensitive and costs were increased to I$2, vaccination alone dominated VIA screening, although VIA combined with vaccination approximated I$975 per YLS (see Supplementary Appendix).

The choice between specific screening modalities was sensitive to assumptions about their relative costs, test performance, and ability to be delivered within one or two visits. For example, if training costs associated with VIA raised the total screening cost such that it would approach that of HPV DNA testing, vaccination plus screening three times per lifetime with two-visit HPV DNA testing was favored, at I$1780 per YLS – if a rapid HPV DNA test was available, allowing for a single-visit screen-and-treat strategy, this result would become even more favourable. All strategies requiring multiple visits became more attractive when loss to follow-up was reduced.

**Figure 3:**
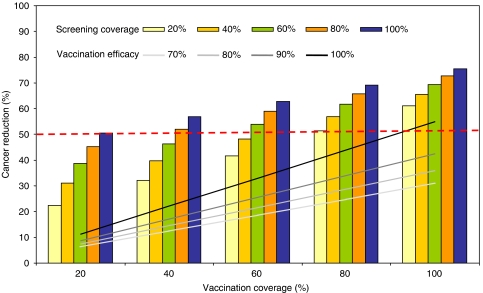
Impact of vaccination coverage, screening coverage, and vaccine efficacy on clinical benefits. This figure depicts how cancer reduction is influenced by different levels of vaccination and screening coverage with a combined strategy of vaccination plus screening three times per lifetime using two-visit HPV DNA testing. Cancer reduction is on the *y* axis, and vaccination coverage on the *x* axis. The coloured bars represent different coverage levels for screening (pale yellow, 20%; gold, 40%; green, 60%; orange, 80%; blue, 100%). The lines represent a strategy of vaccination alone at different levels of vaccine efficacy (white, 70%; light grey, 80%; dark grey, 90%; black, 100%). The dashed red line represents a threshold of 50% cancer reduction.

We have previously reported how the comparative performance of different cervical cancer prevention strategies depends on several factors (Goldie *et al*, 2005, 2007; Garnett *et al*, 2006; Kim *et al*, 2007a). For screening, as noted above, these include test performance, cost, and loss to follow-up. For vaccination, these include vaccine efficacy, coverage, and duration of protection. Figure 3 shows how different assumptions about vaccination and screening coverage will influence the level of cancer reduction achievable with a strategy of pre-adolescent vaccination, assuming 100% efficacy, combined with screening three times per lifetime using two-visit HPV DNA testing (shown by the bars). Similarly, Figure 3 shows how vaccine efficacy and coverage will influence the level of cancer reduction with a strategy of vaccination alone (shown by the lines). A combined strategy of vaccination and screening always provided greater cancer reduction than vaccination alone; however, the incremental benefits achieved by adding screening three times per lifetime were greater at lower vaccination coverage rates.

We can also obtain information about the coverage levels of vaccination and screening required to achieve a specific threshold reduction in cervical cancer from Figure 3. For example, without screening, a threshold of 50% cancer reduction (dashed red line) was expected to be attainable with vaccination alone only at vaccination coverage rates exceeding 80% and vaccine efficacy of 100%. When screening and vaccination were combined, however, several strategies would potentially achieve this threshold. At vaccination coverage rates of 40–50%, screening coverage would need to exceed 80%, but at vaccination coverage of 70%, screening coverage of 40–50% would suffice. Assuming a cost per vaccinated girl of I$10, the vast majority of these combination strategies had cost-effectiveness ratios less than the per capita GDP when compared to the next best strategy.

## Discussion

The vaccine-preventable cervical cancer burden in India is a product of several factors, including the underlying cervical cancer incidence, the proportion of cancer attributable to HPV 16 and 18, the long-term vaccine efficacy, and the ability to achieve high coverage in adolescent girls before sexual activity. Our results showed that with 70% coverage, the expected mean reduction in the lifetime risk of cervical cancer with pre-adolescent vaccination alone was 44%.

The effectiveness of the vaccine could be lower than we projected if older girls, who may have been infected previously with type 16 or 18, are vaccinated; if there is less robust vaccine-induced immunity in girls with other diseases, such as severe anaemia, chronic illness or HIV; or if vaccine-induced immunity wanes while individuals are still at risk for new HPV infections. On the other hand, the impact of the vaccine could be higher if there is long-term cross-protection against non-16,18 type infection; if there are herd immunity benefits to unvaccinated individuals; and if non-cervical HPV 16,18-related cancers and diseases are prevented. For most of these factors, a lack of data prohibit us from estimating the magnitude of individual potential effects; that being said, these are uncertain issues for which empiric data are needed.

We found that a combined approach of pre-adolescent vaccination and screening three times per lifetime after age 30, both at 70% coverage, provided a mean cancer reduction of 56–63%, depending on the specific screening strategy. Because the mechanisms of effectiveness for vaccination and screening differ, with vaccination preventing infections with HPV types 16 and 18, and screening allowing for treatment of precancerous lesions (caused by any high-risk HPV type) before progression to invasive cancer, they are synergistic, and provide substantially greater benefits than either alone. Although screening three time per lifetime prevents additional deaths from non-16 and 18-type associated cancers, as well as 16,18-associated cancers in the proportion of the population not vaccinated, it also provides some insurance of cancer risk protection in the context of the uncertainty around long-term vaccine performance in a non-clinical trial situation. For example, across the good-fitting parameter sets, the mean cancer reduction with vaccination alone ranged from 28 to 57%, whereas with combined vaccination and screening three times per lifetime using two-visit HPV DNA testing ranged from 53 to 76%.

The cost-effectiveness of HPV vaccination will depend mostly on the incremental programmatic costs associated with adding a pre-adolescent vaccine to India's vaccination programme, and also on the ultimate negotiated vaccine price for India. We found that provided the cost per vaccinated girl was I$10 or less (per dose cost of approximately $2), vaccination alone was more effective and cost-effective than screening alone. A combined approach of pre-adolescent vaccination and screening three times per lifetime (at ages 35, 40, and 45) using VIA cost I$290 per YLS; this ratio is a fraction of India's per capita GDP, and would be considered very cost-effective according to suggested benchmarks for developing countries (WHO, 2001). As the cost per vaccinated girl exceeded I$10, vaccination alone was no longer more efficient than screening alone. The incremental cost-effectiveness ratio for pre-adolescent vaccination followed by screening in adulthood three times per lifetime varied from I$340 per YLS at I$20 per vaccinated girl, to I$1920 per YLS at I$75 per vaccinated girl. At a cost per dose of approximately $100 (I$360 per vaccinated girl), vaccination followed by screening exceeded I$7000 per YLS.

Although the rank-ordering of screening strategies from most effective to least effective, was two-visit HPV DNA testing, single-visit VIA, and three-visit cytology, differences between these screening approaches were somewhat attenuated at higher vaccination coverage rates. Vaccination plus screening three times per lifetime with two-visit HPV DNA screening consistently exceeded I$80 000 per YLS due to a VIA strategy's lower cost and ability to deliver screening and treatment in a single visit. However, with plausible changes in relative costs and test performance, and the ability to obtain a same-day test result, the use of HPV DNA testing was equally attractive. If a cytology-based strategy could be conducted in two visits without the use of colposcopy for diagnostic confirmation, it could be more attractive than predicted by our analysis due to reduced loss to follow-up. The results of this sensitivity analysis indicate that regions in India should consider their own situations and infrastructure, and identify which of these screening test options will be most feasible for them. Most importantly, regardless of specific test choice, our results provide strong support for screening adult women two to three times per lifetime in addition to pre-adolescent vaccination.

Other influential assumptions on the cost-effectiveness ratio include the values used for vaccine efficacy and the annual discount rate. If vaccine efficacy is low, girls who are not protected still accrue the costs of vaccination, thereby making it less cost-effective. Cost-effectiveness analyses should be revisited as future data become available on efficacy, necessity for and cost of boosters, and ability to reach young girls who are not yet sexually active. Since the cancer prevention benefits to vaccinated girls occur years after the costs are paid, the effect of discounting both costs and benefits equally, as recommended by guidelines for economic evaluations, (DCPP; Gold *et al*, 1996; Drummond *et al*, 2005; WHO CHOICE) is substantial. We acknowledge the complexity of the decision faced by countries on whether to adopt the HPV vaccine, given the irrefutable reality that women who will benefit from vaccination are not in the same birth cohorts as the women who benefit from screening in the short-run. Even so, as we have documented in other work, the benefits of HPV 16,18 vaccination, when presented in formats that are not influenced by discounting, are comparable to those of other new vaccines. For example, we found that per 1000 girls vaccinated, 15 deaths would be averted (Goldie *et al*. 2008 in press), which compares favourably to 3 deaths prevented per 1000 children vaccinated for rotavirus (IAVI/PATH, 2007).

We recognise that because vaccination and screening are applied to such different age groups, rely to different degrees on existing infrastructure, and require the mobilisation of financial resources that are likely to come from different ‘pots’, the feasibility of achieving wide coverage with screening *vs* vaccination could vary greatly within India, providing a challenge in designing a national prevention programme. It is possible that in regions where screening is not likely to be feasible, focused efforts to achieve high coverage rates for pre-adolescent vaccination would be the most worthwhile investment. In contrast, in regions where screening is successfully being conducted within demonstration projects or clinical studies, it may be most cost-effective to continue to expand those efforts and combine them with pre-adolescent vaccination – at vaccination coverage rates of even 50%, a combined approach could substantially reduce the incidence of cancer.

In India, where out-of-pocket costs of health care can topple a household into extreme poverty, if the opportunity for vaccination is restricted to those willing to pay higher costs in the private sector, most women will not benefit. From a population perspective, since 80% of Indian women live on less than I$2 per day (World Bank), even at a drastically reduced price, the vast majority could not afford this vaccine and provision by the public health sector will be necessary. Although a cost-effectiveness analysis provides information on value for money to a decision maker with a long-term perspective on investing in health, it is not equivalent to providing information on affordability to the payer for whom the short-term perspective is more relevant. Both the financial costs (i.e., affordability) and the cost-effectiveness profile (i.e., value for money) of an HPV vaccine will need to be favourable as this vaccine will compete for dollars earmarked for existing immunisation programs.

Our analysis has several limitations, and thus, we emphasise that our results are intended to provide quantitative approximations of the potential benefits of HPV vaccination, and to provide qualitative insight into the relative value of primary and secondary prevention. We have previously discussed the limitations inherent in our modelling approach (Goldhaber-Fiebert *et al*, 2007; Goldie *et al*, 2007; Kim *et al*, 2007a), but we briefly mention key points here. In addition to model parameter uncertainty, there are uncertainties with respect to the natural history of HPV, especially in older women, the nature of type-specific immunity following natural infection, and the relationships between different HPV types in the case of multiple infections. Similarly, a lack of high quality data on temporal trends of HPV-related disease limited our ability to calibrate the model to different time points, and contributed to the choice to simulate a single birth cohort.

We made a tradeoff in choosing to use our individual-based microsimulation model for this analysis and not our HPV 16 and 18 transmission model (Kim *et al*, 2007b). This was a purposeful decision as the former can simulate detailed screening strategies as well as vaccination, and includes other HPV types not targeted by the vaccine, enabling us to take advantage of all available data by calibrating to many epidemiological targets (Garnett *et al*, 2006). As the model includes vaccine-targeted and non-targeted HPV types, we were able to explore the potential effect of an increase in non-vaccine-targeted HPV types. We have previously documented a small expected increase in non-16,18 cancers (Goldie *et al*, 2003, 2007), although we again found that the impact on the main cost-effectiveness results was small. While our independent dynamic model (Kim *et al*, 2007b) has been previously used to include the herd immunity effects in an HPV vaccine policy analysis, sensitivity analyses that reduced incidence based on these findings, found the main policy results would be unlikely to change. Data on type-specific transmission by age and sex will be useful to include in future analyses once available.

Given the range of uncertainties and the limitations of this analysis, our findings should be considered exploratory and our estimates of cost-effectiveness approximate, and should be interpreted in the context of the analytic purpose of this work, that is, to leverage the best available data to provide insight into decisions that policy makers in India are discussing right now. A country as large and as heterogeneous as India will need to conduct its own financial analyses, assess the effectiveness and feasibility of alternative modes of delivery, and identify potential economies of scale with other programmes that might target adolescents. The strategies that we have identified as cost-effective in this analysis may still be prohibitively expensive in India, and information on affordability, potential financing mechanisms, and likelihood of uptake and acceptability will need to be considered by decision makers as well.

Prevention and treatment of cervical cancer, the leading cause of cancer in Indian women, is a priority according to the country's National Cancer Control Programme (Government of India, 2005). On the basis of age-specific incidence and projected demographic changes, the expected new cases of cervical cancer in India will increase from 132 082 in year 2002, to more than 330 000 in 2050 (Ferlay *et al*, 2004). The vast majority of women will lack curative treatment, and thus, approximately two-thirds will die from this preventable disease. The implications for these women's families and communities are profound, as this cancer affects women at an age when they are vital to social and economic stability. The opportunity to prevent these deaths is now inarguable with the availability of a vaccine to prevent HPV 16,18 infection, new diagnostics for HPV DNA testing, and promising secondary prevention strategies that target women after age 30 and perform screening and treatment in as few visits as possible (Goldie *et al*, 2005). If the cost per vaccinated girl is less than I$10, implying a per dose cost of approximately $2, vaccination is likely to be extremely cost-effective in India. The most effective strategy, within a framework that would still be potentially cost-effective in India, would be pre-adolescent vaccination, followed by screening three times per lifetime between ages 35 and 45; assuming a vaccination coverage rate of 70%, this strategy would be expected to prevent more than 1.25 million cervical cancer deaths over the lifetimes of 10 consecutive birth cohorts.

## Figures and Tables

**Table 1:** Selected cost variables^a^

**Variable**	**Base case**
*Vaccine costs* b	
Cost per vaccinated individual	50.00
Vaccine cost (three doses × unit cost)	36.74
Vaccine wastage	5.51
Freight, supplies, supply wastage, and administration	2.81
Monitoring and programmatic services	2.94
Cold chain, injection safety, operational costs	2.00
	
*Screening, diagnostic, and treatment costs* c ^,^ d	
HPV DNA test	10.30
Cytology	3.69
Visual inspection with acetic acid	1.25
Colposcopy and biopsy	40.30
Cryotherapy	16.00
Loop electrosurgical excision procedure	106.99
Cold knife conisation	237.02
Simple hysterectomy	338.59
	
*Invasive cervical cancer costs* e	
Local	1611.57
Regional/distant	2346.94
	
*Patient time and transportation costs* f	
Patient average hourly wage	0.30
Screening visit	0.74
Diagnostic visit	15.44
Cryotherapy visit	0.76
Loop electrosurgical excision procedure visit	15.49
Cold knife conisation visit	24.57
Simple hysterectomy visit	37.29

a Costs reported in I$2005, a currency that provides a means of translating and comparing costs among countries, taking into account differences in purchasing power (WHO, 2001). We capitalised on data published previously for an analysis of screening alternatives in India (Goldie *et al*, 2005).

b Vaccine cost is expressed as a composite estimate of cost per vaccinated girl, and this total value is varied from I$5 to I$360 in sensitivity analysis; shown is the base case value. We assume the ‘cost per vaccinated girl’ includes three doses, vaccine wastage, freight into the country, supplies and administration, incremental programmatic costs for immunisation services, and incremental costs of social mobilisation and outreach for a new pre-adolescent vaccine (see Supplementary Appendix).

c Screening costs include staff time, supplies, HPV DNA assay or Papanicolau test, and specimen transport. Diagnostic and treatment costs include staff time, supplies, and equipment depreciation; treatment includes cost of follow-up visits and complications. The cost of the HPV DNA test (i.e., hybrid capture test) was based on a previous analysis (Goldie *et al*, 2005). As this cost was intended to reflect an eventual negotiated price for developing countries, we assumed the same cost for the rapid test and decreased it by 50% in sensitivity analysis.

d Model parameters were varied ±75% in sensitivity analysis.

e Invasive cancer costs include both direct medical and direct non-medical costs. Direct medical costs of cancer care include staging of cancer severity, hospitalisation, stage-appropriate treatment, and follow-up visits. Direct non-medical costs and time costs associated with cancer care include all patient time in transport, waiting, receiving treatment, and hospitalisation as well as actual transport costs.

f Non-medical costs include the time costs for two-way travel, waiting at the clinical site, and receiving treatment, and the cost of transport for an average of two follow-up visits. Screening and cryotherapy visits are carried out at a primary health clinic, whereas all other visits occur at a district hospital (see Supplementary Appendix). HPV, human papillomavirus.

**Table 2:** Mean cancer reduction and impact of the cost per vaccinated girl on the incremental cost-effectiveness ratios (I$/YLS) of cervical cancer prevention strategies^a^

			**Cost per vaccinated girl** b					
			**I$10**	**I$20**	**I$30**	**I$50**	**I$75**	**I$360**
		**Mean cancer**	**Approximate implied per dose cost**					
	**Test**	**reduction**	**US$2**	**US$4**	**US$6**	**US$12**	**US$20**	**US$100**
**Table 2a. All strategies available**								
Natural history (no screening or vaccination)			—	—	—	—	—	—
								
Screening 1 time per lifetime at age 40	One-visit VIA	13%	dom^c^	dom^c^	dom^c^	dom^c^	dom^c^	dom^c^
	Two-visit HPV DNA	16%	dom^c^	dom^c^	dom^c^	dom^c^	dom^c^	dom^c^
								
Screening two times per lifetime at	One-visit VIA	21%	dom^c^	dom^c^	dom^c^	dom^c^	dom^c^	dom^c^
ages 35 and 40	Two-visit HPV DNA	24%	dom^c^	dom^c^	dom^c^	dom^c^	dom^c^	dom^c^
	Three-visit cytology	21%	dom^c^	dom^c^	dom^c^	dom^c^	dom^c^	dom^c^
								
Screening three times per lifetime at	One-visit VIA	29%	dom^c^	**I$60**	**I$60**	**I$60**	**I$60**	**I$60**
ages 35, 40, and 45	Two-visit HPV DNA	33%	dom^c^	dom^c^	dom^c^	dom^c^	dom^c^	dom^c^
								
Vaccination		44%	**CS** d	dom^c^	dom^c^	dom^c^	dom^c^	dom^c^
								
Vaccination+screening one time per	One-visit VIA	52%	dom^c^	dom^c^	dom^c^	dom^c^	dom^c^	dom^c^
lifetime at age 40	Two-visit HPV DNA	53%	dom^c^	dom^c^	dom^c^	dom^c^	dom^c^	dom^c^
								
Vaccination+screening two times per	One-visit VIA	56%	dom^c^	dom^c^	dom^c^	dom^c^	dom^c^	dom^c^
lifetime at ages 35 and 40	Two-visit HPV DNA	57%	dom^c^	dom^c^	dom^c^	dom^c^	dom^c^	dom^c^
	Three-visit cytology	56%	dom^c^	dom^c^	dom^c^	dom^c^	dom^c^	dom^c^
								
Vaccination+screening three times per	One-visit VIA	61%	**I$290**	**I$340**	**I$630**	**I$1200**	**I$1920**	**I$7230**
lifetime at ages 35, 40, and 45	Two-visit HPV DNA	63%	**I$82 540**	**I$82 540**	**I$82 540**	**I$82 540**	**I$82 540**	**I$82 540**
								
**Table 2b. Only two-visit HPV DNA testing available**								
Natural history			—	—	—	—	—	—
Screening alone three times per lifetime			domc	domc	domc	**I$720**	**I$720**	**I$720**
Vaccination			**CS** d	**I$190**	**I$390**	**I$890**	domc	domc
Vaccination+screening three times per lifetime			**I$1780**	**I$1780**	**I$1780**	**I$1780**	**I$2030**	**I$7650**

a After eliminating strategies that are dominated, incremental cost-effectiveness ratios are calculated for the remaining strategies and are expressed in I$2005 per YLS. The incremental cost-effectiveness ratios shown represent the mean costs divided by the mean effects of the good-fitting parameter sets.

b Cost per vaccinated girl includes three doses of vaccine, wastage, freight and supplies, administration, and immunisation support and programmatic costs.

c dom: these strategies are either more costly and less effective, or less costly and less cost-effective, than alternative options, and are thus considered dominated.

d CS: cost saving.

HPV: human papillomavirus.

VIA: visual inspection with acetic acid.
