# Supplementary material for: Health and economic impact of HPV 16 and 18 vaccination and cervical cancer screening in India
Source: Br J Cancer. 2008 Jul 8;99(2):230–8. doi: 10.1038/sj.bjc.6604462 (PMC2480962; doi:10.1038/sj.bjc.6604462)
Supplement: Supplementary Appendix [file 6604462x1.pdf]

SUPPLEMENTARY APPENDIX

Accompanying the manuscript:

HEALTH AND ECONOMIC IMPACT OF HPV 16 AND 18 VACCINATION AND CERVICAL CANCER SCREENING IN INDIA

Mireia Diaz, Jane J. Kim, Ginesa Albero, Silvia de Sanjosé, Gary Clifford, F. Xavier Bosch, and Sue J. Goldie

This Technical Appendix provides selected assumptions and methods for our model of human papillomavirus and cervical cancer, including the model parameterization process, screening strategies, cost estimates, and results not available in the main paper.

Part I: Model parameterization

Overview

Because many of the processes of HPV infection, progression, and clearance are uncertain, the model was calibrated to accurately match observed epidemiologic data of the natural history of HPV infection and cervical cancer. Model calibration determined combinations of model inputs (i.e., “input parameter sets”) that produce model outcomes simultaneously consistent with epidemiological data from multiple sources (i.e., “calibration targets”). Multiple input parameter sets were randomly generated, used in the model to produce model output, and evaluated in terms of how well the model outputs simultaneously fit the calibration targets (Kim 2007).

Model input parameters: prior distributions and searching

Our calibration methods have been documented elsewhere (Kim 2007), but briefly, a comprehensive literature review on epidemiologic data was conducted to define baseline age-specific natural history model inputs and plausible ranges around model parameters. We assumed the basic relationship between infection with high-risk types of HPV and cervical carcinogenesis does not fundamentally differ between countries and therefore our initial plausible ranges were based on the best available data regardless of setting. However, given that the epidemiology, risk factors, and burden of disease vary considerably between countries, we calibrated the model to India-specific epidemiological data, which included age- and type-specific HPV prevalence, age-specific prevalence of cervical intraepithelial lesions, HPV type distribution within different grades of cervical cancer, and age-specific cancer incidence. We elected to adapt the model to Southeastern India based on the availability of data for nearly all epidemiological targets required for our calibration procedure (Franceschi 2003, 2005, Parkin 2005). We searched over ranges of multipliers that were applied to these baseline inputs. The baseline inputs and multipliers together encompassed a uniform joint prior distribution on model input parameters.

Input parameter sets were drawn from the joint prior distribution. Multiple simulations of the natural history model were conducted for populations of 100,000 individuals. For a single simulation, one value for each parameter was randomly selected from a uniform distribution over the identified range. In total, simulations were conducted with 555,000 uniquely sampled parameter sets. Model outcomes (e.g., age-specific prevalence of CIN1 or HPV type-distribution in invasive cervical cancer) using each parameter set were compared with multiple epidemiologic targets using a likelihood-based approach.

Defining calibration targets

In total, 64 calibrations targets were defined. Calibration targets included type- and age-specific prevalence of HPV, age-specific prevalence of CIN categories, type distribution within CIN categories, age-specific cancer incidence, cumulative cancer incidence, and type- and age-

specific duration of HPV infections and CIN. For each calibration target, we determined a point estimate and confidence interval, using population-based data sources.

Calibration target data were not used directly in the initial model parameterization; instead they informed multipliers of these initial model inputs. All prevalence and HPV type distribution targets were calculated using 95% confidence intervals of the binomial distribution in STATA/SE 9.0 (Reiczigel 2003, Tobi 2005). Cancer incidence targets were determined using the lower limit of the 95% confidence interval of the minimum rates from Bangalore registries as the lower bound, and the upper limit of the 95% confidence interval of the maximum rates from Chennai registries as the upper bound. The 95% confidence interval was calculated using the standard error for a rate (NY State Dept of Health).

Goodness-of-fit

The model outputs from each input parameter set were compared to the calibration targets. Model fit to the targets was evaluated by constructing a goodness-of-fit score. A composite goodness-of-fit score for each parameter set was computed by summing the log likelihood of each model outcome (Kim 2007). Goodness-of-fit scores followed a chi square distribution with the number of degrees of freedom equal to the number of targets.

Input parameter acceptance criterion

Our acceptance criterion had two parts: 1) overall goodness-of-fit; and 2) emphasis of population targets of greatest importance for policy questions relating to HPV vaccination and testing.

First, we determined our best-fitting parameter set as the one with the lowest goodness-of-fit score – the model-generated input parameter whose simulated model outputs were simultaneously closest to all calibration targets. We identified parameter sets that were statistically indistinguishable from the best-fitting set. To do so, we calculated a critical goodness-of-fit value. We used a likelihood ratio test with the null hypothesis being that the critical goodness-of-fit score is equal to our best goodness-of-fit score. The alternative hypothesis is that they are not equal. We fixed our significance level at p=0.05 and then identified the lowest goodness-of-fit score for which we could reject the null hypothesis at this significance level. All parameter sets with goodness-of-fit scores greater than this critical value were discarded, and all others (for which we could not reject the null hypothesis of having goodness-of-fit scores equal to the best fitting parameter set's goodness-of-fit score) were retained and considered "good fitting".

Good-fitting input parameter set selection for cost-effectiveness analyses

Because of the potentially large number of parameter sets retained through this acceptance rule, we sampled from the accepted input parameter sets to generate a representative subset of the best parameter sets for use in our cost-effectiveness analysis. To incorporate the effect of parameter uncertainty, cost-effectiveness analyses were conducted with a selected sample of

good-fitting parameter sets, and results were reported as the mean and range of outcomes, while incremental cost-effectiveness ratios were reported as the ratio of the mean costs divided by the mean effects of a sample of good-fitting parameter sets (Stinnett 1997).

Calibration target data sources

Age-specific prevalence of high-risk and low-risk HPV types

Population-based epidemiologic data in India show variation in HPV prevalence. Based on data from 17,365 women with normal cytology, HPV prevalence ranged from 4.8% in Trivandrum to 7.8% in Kolkata (Sankaranarayanan 2004). An HPV survey conducted by the International Agency for Research (IARC) in a rural area of Tamil Nadu, a southern India state, reported an HPV prevalence of 14% using a PCR-based HPV assay, based on 1,799 women with normal cytology (Franceschi 2005). HPV prevalence of women attending screening programs in three states of northeast India ranged from 6.7% in Manipur to over 11% in Sikkim and West Bengal (Laikangbam 2007). Although the two most common types detected in invasive cervical cancer are HPV 16 (55%) and HPV 18 (15%) (Smith 2007), studies in India have reported HPV 16 in 53.5% to 73.6% of cases, and HPV 18 in 9.3% to 17.1% (Bhatla 2006, Franceschi 2003, Munirajan 1998, Peedicayil 2006, Sowjanya 2005).

IARC survey data from women enrolled in a study in the Dindigul District of Tamil Nadu were used to inform the age-specific prevalence of high-risk and low-risk HPV types, CIN 1 and CIN 2,3 (Franceschi 2005). The survey was conducted between February and October 2003 in 109 panchayats (local administrative structures consisting of 4-23 villages) and invited a total of 2,000 married, non-pregnant women aged 16-59 years. Data collected from women with adequate HPV test results and normal cytology were used to inform our HPV type prevalence estimates. The HPV prevalence in women with normal cytology was 14.0 (252 out of 1,799) and the prevalence of cervical lesions was 5.0% (94 out of 1,983). No cases of cancer were observed.

Table 1. Age-specific prevalence of HR and LR HPV types in *women with normal cytology* and 95% confidence intervals.

Age Group	N Total [a]	HPV-HR				HPV-LR			
		N	Prevalence	95% CI		N	Prevalence	95% CI	
**12-14**	-	-				-	-		
**15-19**	24	3	0.125	0.0266	0.3236	2	0.083	0.0103	0.2700
**20-24**	297	25	0.084	0.0552	0.1218	15	0.051	0.0285	0.0819
**25-29**	566	50	0.088	0.0663	0.1148	22	0.039	0.0245	0.0583
**30-34**	238	22	0.092	0.0588	0.1366	10	0.042	0.0203	0.0759
**35-39**	256	28	0.109	0.0739	0.1542	10	0.039	0.0391	0.0707
**40-44**	168	21	0.125	0.0791	0.1847	5	0.03	0.0097	0.0681
**45-49**	141	12	0.085	0.0448	0.1439	6	0.043	0.0158	0.0903
**50-54**	74	8	0.108	0.0478	0.2020	3	0.041	0.0084	0.1139
**55-59**	35	3	0.086	0.0180	0.2306	1	0.029	0.0007	0.1492
**60-64**	-	-				-			
**65-69**	-	-				-			

**70-74**	-	-				-			
**>74**	-	-				-			
	**1,799**	**172**	0.096			**74**	0.041		

[a] Total number includes those women with normal cytology and adequate HPV test result

Age-specific prevalence of CIN 1 and CIN 2,3

Table 2. Age-specific *prevalence of CIN1 and CIN2-3* and 95% confidence intervals.

Age Group	N Total [a]	CIN 1				CIN 2-3			
		N	Prevalence	95% CI		N	Prevalence	95% CI	
**12-14**	-	-				-			
**15-19**	24	0	0.0000	0.0000	0.1425	0	0.0000	0.0000	0.1425
**20-24**	311	14	0.0450	0.0248	0.0976	0	0.0000	0.0000	0.0118
**25-29**	593	22	0.0371	0.0234	0.0734	5	0.0084	0.0027	0.0242
**30-34**	254	13	0.0512	0.0275	0.1097	3	0.0118	0.0024	0.0398
**35-39**	268	9	0.0336	0.0155	0.0815	3	0.0112	0.0023	0.0378
**40-44**	178	7	0.0393	0.0160	0.1009	3	0.0169	0.0035	0.0565
**45-49**	151	6	0.0397	0.0147	0.1017	4	0.0265	0.0073	0.0845
**50-54**	78	1	0.0128	0.0003	0.0694	3	0.0385	0.0080	0.1261
**55-59**	36	1	0.0278	0.0007	0.1453	0	0.0000	0.0000	0.0974
**60-64**	-	-				-			
**65-69**	-	-				-			
**70-74**	-	-				-			
**>74**	-	-				-			
	**1,893**	**73**	0.0386			**21**	0.0111		

[a] Total number includes those women who did not have an adequate HPV test result, but did have adequate cytology.

HPV type prevalence by lesion status

We estimated the percentages of HPV 16, HPV 18, or high risk other in CIN 2,3 and the percentage of HPV16/18 or high risk other in CIN 1 based on the IARC HPV prevalence survey (Franceschi 2005). The percentages of HPV 16 and HPV 18 in invasive cervical cancer cases were estimated from an international multicenter case-control study of invasive cervical cancer (Franceschi 2003). This study was conducted in the Cancer Institute in Chennai from June 1998 to May 1999 with 222 women diagnosed with invasive cervical cancer; control women were other inpatients or visitors at the Cancer Institute.

Table 3. HPV type prevalence by lesion and 95% confidence intervals.

Lesion Category & HPV Type	N total [a]	N HPV-type specific	Prevalence	95% CI	
**CIN1 [b]**					
**HPV 16 & 18**	72	16	0.2222	0.1327	0.3356
**HR Other**	72	32	0.4444	0.3272	0.5664
**CIN2-3 [b]**					
**HPV 16**	20	7	0.3500	0.1539	0.5922
**HPV 18**	20	2	0.1000	0.0123	0.3170
**HR Other**	20	8	0.4000	0.1912	0.6395
**CANCER [b]**					
**HPV 16**	191	120	0.6283	0.5555	0.6969
**HPV 18**	191	28	0.1466	0.0997	0.2049

[a] Total number includes those women with adequate HPV test result.

[b] Includes 21 HPV negative women for CIN1, 3 for CIN 2-3, and 1 for invasive cancer

Age-specific cervical cancer incidence

There are ten unique cancer registries in India included in Cancer Incidence in Five Continents (Parkin 2005):

- Ahmedabad (1983-1987, 1993-1997)
- Bangalore (1982, 1983-1987, 1988-1992, 1993-1997)
- Barshi (1988-1992)
- Chennai (1982, 1983-1987, 1988-1992, 1993-1997)
- Delhi (1993-1996)
- Karunagappally (1991-1992, 1993-1997)
- Mumbai (1964-1966, 1968-1972, 1973-1975, 1978-1982, 1983-1987, 1988-1992, 1993-1997)
- Nagpur (1980-1982, 1993-1997)
- Poona (1973-1977, 1978-1982, 1993-1997)
- Trivandrum (1991-1992, 1993-1997)

Among these sites, there is great geographical variation for the age-specific rates of cervical cancer incidence (**Figure 1**).

Figure 1. Age-specific cervical cancer incidence rates, India, 1964-1997, various sites.

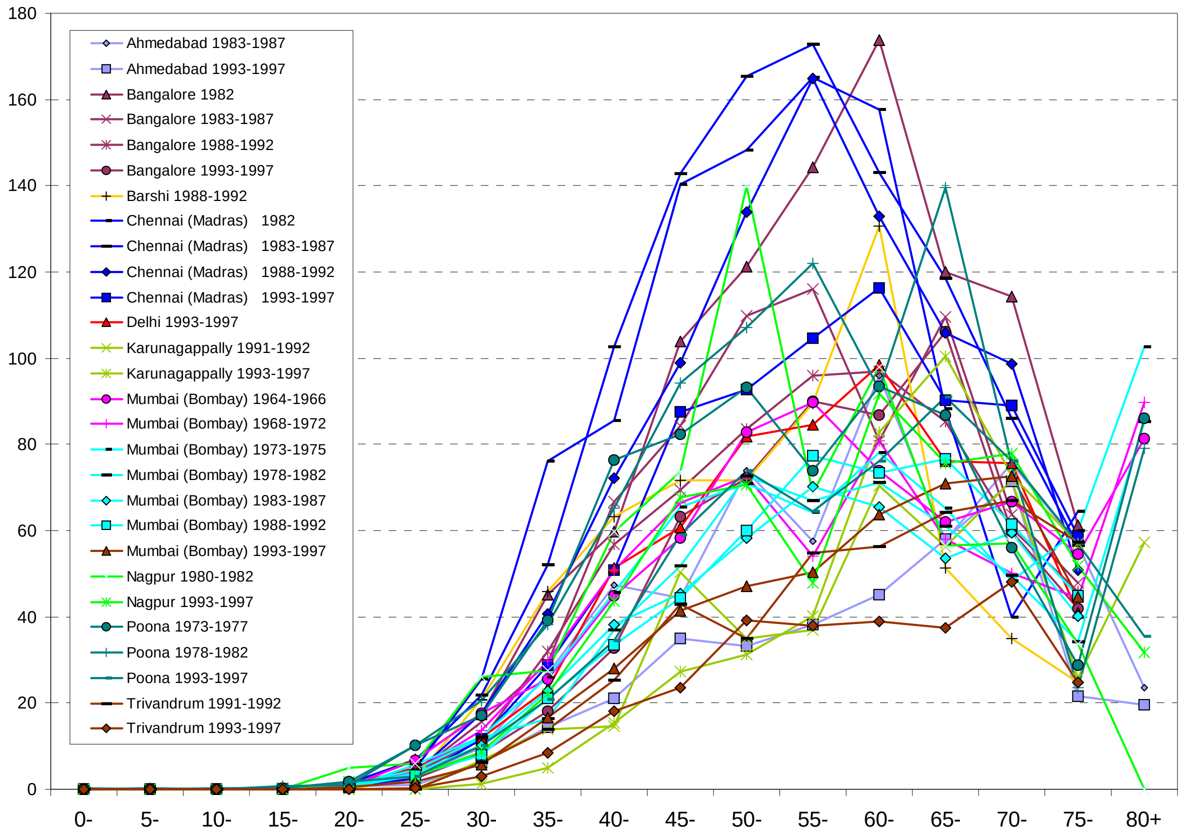

As the studies on HPV prevalence and cervical lesions were from Southeastern India, the Bangalore and Chennai registries were selected to calculate the targets (Tables 4 and 5). These data, with a total of 13,646 cancer cases from 1982 to 1997, provided estimates of the number of incident cases for five-year age ranges (20 to 75+) and the size of population at risk of developing invasive cervical cancer.

Table 4. Cervical cancer incidence data from Bangalore cancer registries (1982-1997).

Period	1982			1983 - 1987			1988 - 1992			1993 - 1997		
**Age Group**	**Population**	**Cases**	**Rate**	**Population**	**Cases**	**Rate**	**Population**	**Cases**	**Rate**	**Population**	**Cases**	**Rate**
**0-4**	197,311	0	0.00	943,540	0	0.00	946,330	0	0.00	1,031,988	0	0.00
**5-9**	204,881	0	0.00	1,059,800	0	0.00	1,166,318	0	0.00	1,247,303	0	0.00
**10-14**	172,381	0	0.00	947,865	0	0.00	1,111,729	0	0.00	1,269,704	0	0.00
**15-19**	152,523	0	0.00	861,655	2	0.23	1,018,904	3	0.29	1,188,322	1	0.08
**20-24**	152,127	2	1.31	892,765	10	1.12	1,079,333	9	0.83	1,272,736	4	0.31
**25-29**	130,519	9	6.90	799,765	30	3.75	1,006,913	40	3.97	1,271,339	31	2.44
**30-34**	92,303	16	17.33	553,050	87	15.73	689,444	79	11.46	927,807	79	8.51
**35-39**	84,031	38	45.22	476,730	140	29.37	564,958	181	32.04	852,930	154	18.06
**40-44**	60,338	36	59.66	330,270	220	66.61	380,657	216	56.74	563,546	185	32.83
**45-49**	47,178	49	103.86	292,840	247	84.35	378,350	263	69.51	463,092	293	63.27
**50-54**	42,046	51	121.30	244,085	268	109.80	300,618	251	83.49	359,511	259	72.04
**55-59**	23,582	34	144.18	156,800	182	116.07	214,506	206	96.03	248,899	224	90.00
**60-64**	27,053	47	173.73	175,610	142	80.86	237,487	230	96.85	269,394	234	86.86
**65-69**	13,338	16	119.96	97,560	107	109.68	142,906	122	85.37	155,612	165	106.03
**70-74**	12,242	14	114.36	80,130	51	63.65	109,130	66	60.48	113,658	68	59.83
**75+**	13,038	8	61.36	83,555	40	47.87	139,210	64	45.97	152,579	64	41.95
**TOTAL**	1,424,891	321	22.53	7,996,020	1,527	19.10	9,486,793	1,732	18.26	11,388,420	1,765	15.50

Table 5. Cervical cancer incidence data from Chennai cancer registries (1982-1997).

**Period**	**1982**			**1983 - 1987**			**1988 - 1992**			**1993 - 1997**		
**Age Group**	**Population**	**Cases**	**Rate**	**Population**	**Cases**	**Rate**	**Population**	**Cases**	**Rate**	**Population**	**Cases**	**Rate**
**0-4**	197,783	0	0.00	865,040	0	0.00	942,683	0	0.00	761,425	0	0.00
**5-9**	205,026	0	0.00	980,050	0	0.00	1,068,013	0	0.00	908,665	0	0.00
**10-14**	180,664	0	0.00	964,330	0	0.00	1,050,881	0	0.00	935,775	0	0.00
**15-19**	163,840	0	0.00	925,720	0	0.00	1,008,808	0	0.00	1,000,595	0	0.00
**20-24**	173,024	2	1.16	926,585	14	1.51	1,009,755	9	0.89	1,119,190	7	0.63
**25-29**	147,730	7	4.74	819,640	55	6.71	893,213	34	3.81	1,042,140	27	2.59
**30-34**	105,957	27	25.48	582,385	127	21.81	634,651	112	17.65	785,175	92	11.72
**35-39**	105,141	80	76.09	552,065	287	51.99	601,616	244	40.56	738,905	212	28.69
**40-44**	76,098	65	85.42	409,655	420	102.53	446,421	322	72.13	547,920	279	50.92
**45-49**	62,680	88	140.40	373,610	534	142.93	407,142	403	98.98	460,230	403	87.56
**50-54**	56,655	84	148.27	302,225	500	165.44	329,363	441	133.89	374,205	347	92.73
**55-59**	37,527	62	165.21	213,625	369	172.73	232,800	384	164.95	306,975	321	104.57
**60-64**	39,292	62	157.79	209,100	299	142.99	227,870	303	132.97	272,640	317	116.27
**65-69**	19,274	17	88.20	122,315	145	118.55	133,294	141	105.78	179,590	162	90.21
**70-74**	15,004	6	39.99	92,955	80	86.06	101,298	100	98.72	122,335	109	89.10
**75+**	12,429	8	64.37	84,740	48	56.64	92,334	47	50.90	139,685	82	58.70
**TOTAL**	1,598,201	518	32.41	8,424,040	2,885	34.25	9,180,142	2,540	27.67	9,695,450	2,358	24.32

Table 6 shows the minimum rates and lower bound from Bangalore, and the maximum rates and upper bound from Chennai.

Table 6. Cervical cancer incidence targets and 95% confidence intervals.

Age Group	Minimum rates [a]	LB [b]	Maximum rates [c]	UB [d]
**15-19**	0,00	0,00	0,00	0,37
**20-24**	0,31	0,09	1,51	2,54
**25-29**	2,44	1,66	6,71	8,73
**30-34**	8,51	6,74	25,48	37,07
**35-39**	18,06	15,32	76,09	94,69
**40-44**	32,83	28,27	102,53	112,81
**45-49**	63,27	56,23	142,93	155,57
**50-54**	72,04	63,54	165,44	180,58
**55-59**	90,00	78,60	172,73	191,27
**60-64**	80,86	68,11	157,79	202,24
**65-69**	85,37	70,90	118,55	139,47
**70-74**	59,83	46,46	98,72	120,06
**75+**	41,95	32,30	64,37	126,79

[a] Minimum rates for Bangalore registries [b] Lower Bound (LB): 95% lower confidence limit of the normal distribution for the minimum rates of the Bangalore registries [c] Maximum rates for Chennai registries [d] Upper Bound (UB): 95% upper confidence limit of the normal distribution for the maximum rates of the Chennai registries

Figure 2. Age-specific cervical cancer incidence rates and 95% confidence intervals.

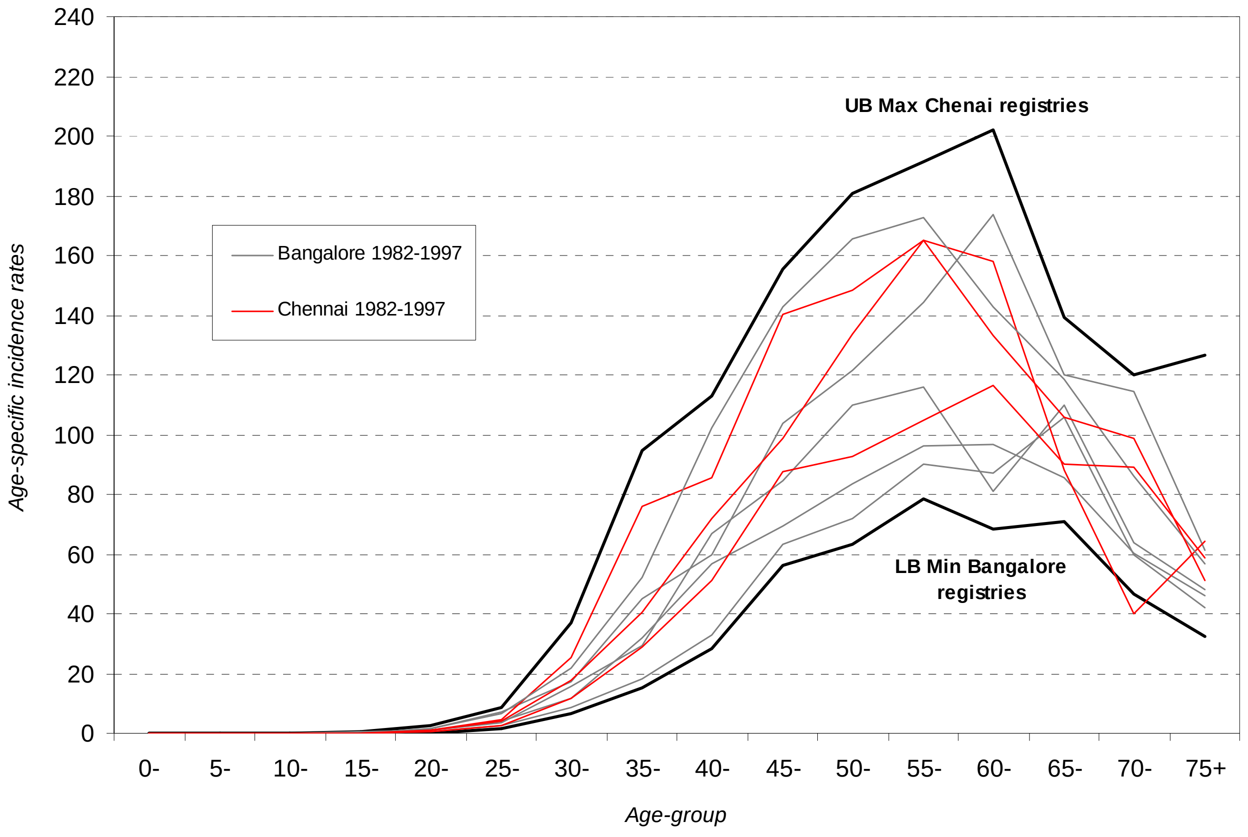

Additional calibration results

The blue line represents the prevalence target, the black heavy solid lines the confidence intervals of the target data at each age group, and the thin lines, the model output for a sample of good-fitting sets. The red lines indicate the 5 best-fitting sets.

Figure 3. Calibration results.

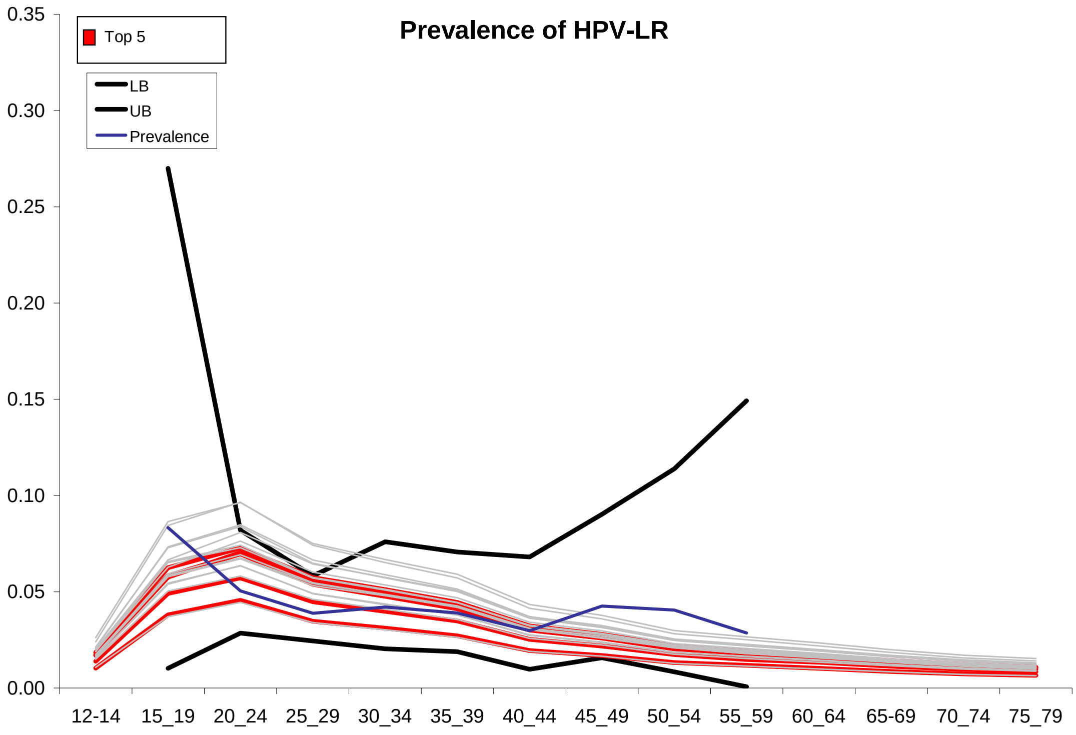

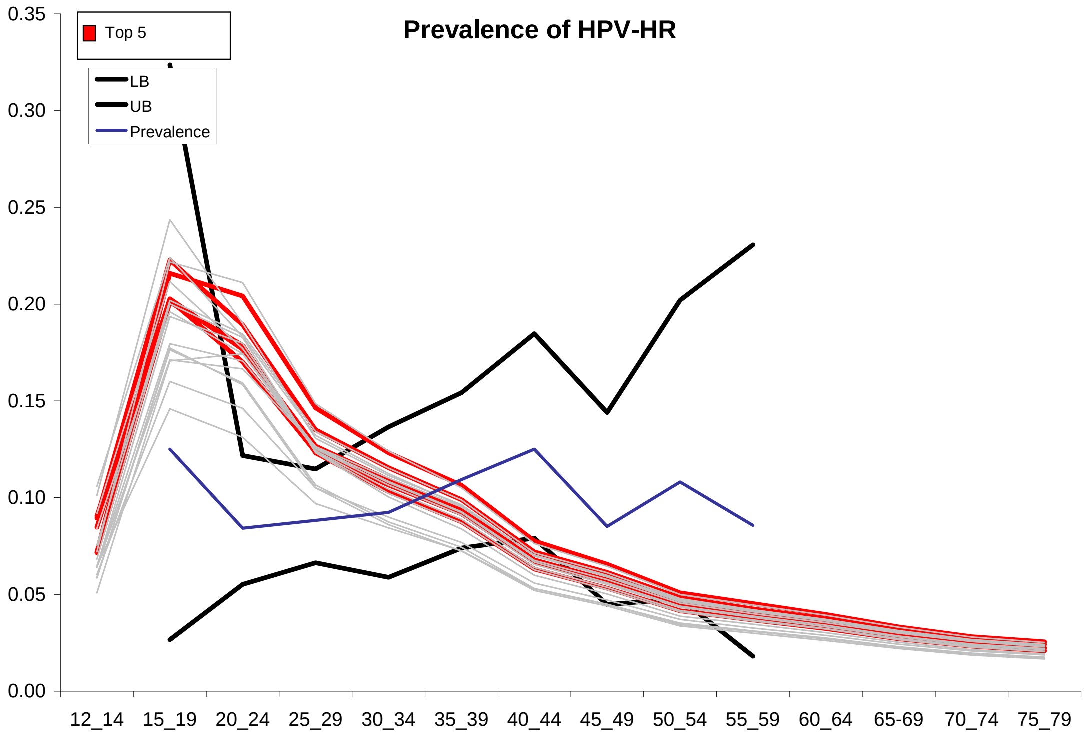

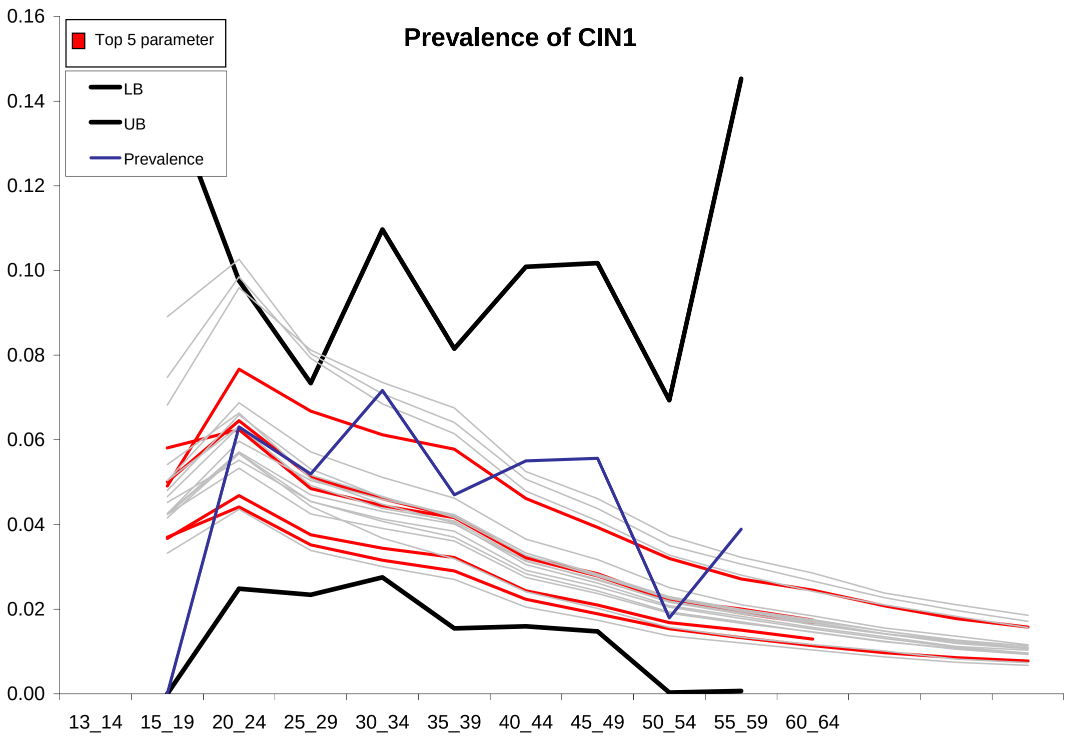
Prevalence of CIN1
Top 5 parameter
LB
UB
Prevalence
0.16
0.14
0.12
0.10
0.08
0.06
0.04
0.02
0.00
13_14
15_19
20_24
25_29
30_34
35_39
40_44
45_49
50_54
55_59
60_64

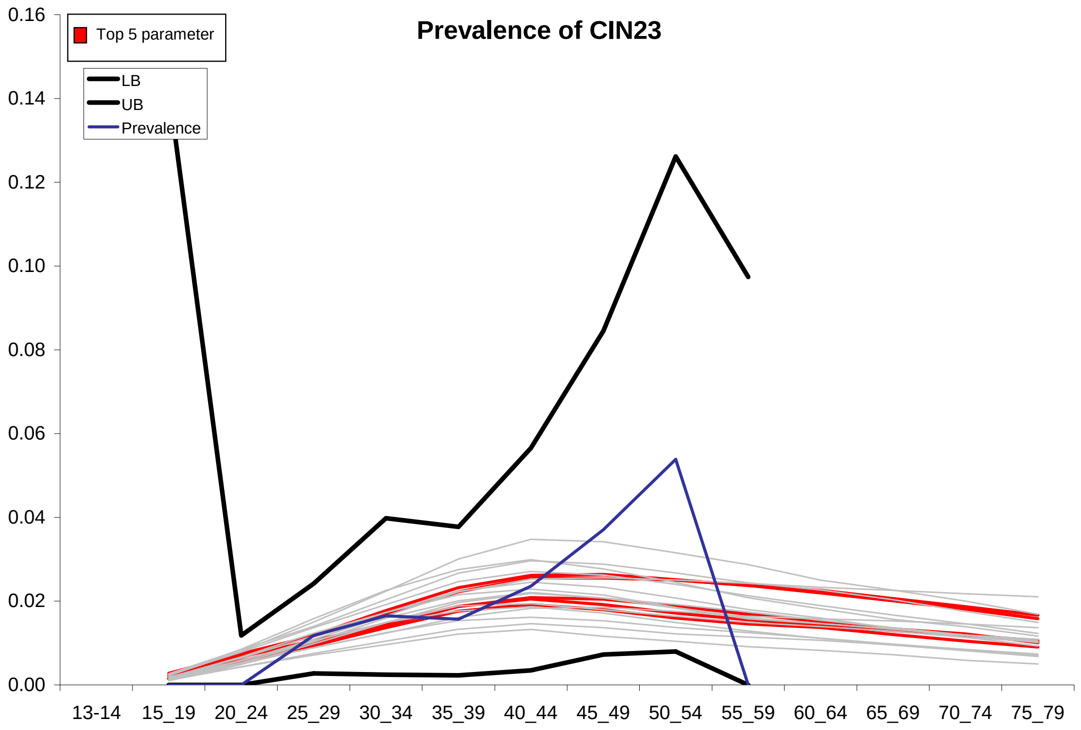
Prevalence of CIN23
Top 5 parameter
LB
UB
Prevalence
0.16
0.14
0.12
0.10
0.08
0.06
0.04
0.02
0.00
13-14
15_19
20_24
25_29
30_34
35_39
40_44
45_49
50_54
55_59
60_64
65_69
70_74
75_79

Part II: Best-Fitting Parameter Sets

Table 7. Posterior input parameters: 'best set' ranges found during calibration. PROGRESSION VARIABLES.

	Normal to HPV DNA			
	**Low-risk (LR) HPV**	**High-risk 16 (HR-16) HPV**	**High-risk 18 (HR-18) HPV**	**High-risk other (HR-other) HPV**
Best Set	2.88104	1.76466	2.91775	4.36633
2nd Best Set	1.78528	4.25022	0.354163	1.48357
3rd Best Set	2.36704	4.66536	4.21737	3.8061
4th Best Set	1.20746	4.6053	7.43338	2.64334
5th Best Set	2.68925	6.33997	4.83463	1.45116
6th Best Set	1.95603	2.62449	2.65843	1.93805
7th Best Set	2.45119	1.4405	7.05544	3.17799
8th Best Set	2.69637	7.29893	1.48026	5.96425
9th Best Set	2.23571	2.69119	0.758845	3.15561
10th Best Set	2.9742	3.34726	5.1054	3.28143
	**HPV DNA to CIN 1**			
	**Low-risk (LR) HPV**	**High-risk 16 (HR-16) HPV**	**High-risk 18 (HR-18) HPV**	**High-risk other (HR-other) HPV**
Best Set	4.65077	1.93784	2.70367	4.15621
2nd Best Set	4.57916	1.92775	4.18929	5.3997
3rd Best Set	1.79759	5.33342	4.22548	4.89646
4th Best Set	2.2752	4.85993	0.652774	5.56885
5th Best Set	2.9678	5.67532	0.563926	5.59714
6th Best Set	3.86646	3.24198	1.65435	5.82736
7th Best Set	5.08197	5.38797	2.37197	5.06321
8th Best Set	3.17133	3.42559	2.51259	5.74408
9th Best Set	3.22213	1.72352	5.59651	5.04381
10th Best Set	2.67431	2.21278	0.536397	4.33308

Table 7. Posterior input parameters: 'best set' ranges found during calibration. PROGRESSION VARIABLES (cont.).

	HPV DNA to CIN 2,3			
	**Low-risk (LR) HPV**	**High-risk 16 (HR-16) HPV**	**High-risk 18 (HR-18) HPV**	**High-risk other (HR-other) HPV**
Best Set	0.024441	0.653378	0.086182	0.008826
2nd Best Set	0.045072	0.319945	0.089672	0.091393
3rd Best Set	0.039071	0.722776	0.094414	0.07469
4th Best Set	0.023463	0.601447	0.064133	0.080354
5th Best Set	0.05886	0.825145	0.050316	0.076024
6th Best Set	0.090318	0.667323	0.067981	0.094215
7th Best Set	0.034071	0.934279	0.03928	0.039152
8th Best Set	0.022712	0.198932	0.009288	0.032766
9th Best Set	0.066522	0.422575	0.07503	0.037474
10th Best Set	0.056169	0.19577	0.051175	0.058701

	CIN1 to CIN 2,3			
	**Low-risk (LR) HPV**	**High-risk 16 (HR-16) HPV**	**High-risk 18 (HR-18) HPV**	**High-risk other (HR-other) HPV**
Best Set	2.46514	5.08882	0.388289	2.88836
2nd Best Set	5.36158	0.612957	5.76637	1.43401
3rd Best Set	4.43231	2.69082	1.03837	2.06579
4th Best Set	0.13277	3.22272	5.91198	0.79442
5th Best Set	5.46802	5.55014	4.06091	0.910731
6th Best Set	0.19343	0.720444	1.44359	1.96269
7th Best Set	0.773888	5.10265	4.78752	1.49257
8th Best Set	2.57864	2.61822	5.21795	1.09856
9th Best Set	1.92802	4.07692	5.33466	0.794285
10th Best Set	5.07268	3.55781	4.70189	1.71291

Table 7. Posterior input parameters: 'best set' ranges found during calibration. PROGRESSION VARIABLES (cont.).

	CIN 2,3 to local cancer		
	**High-risk 16 (HR-16) HPV**	**High-risk 18 (HR-18) HPV**	**High-risk other (HR-other) HPV**
Best Set	2.15534	5.26901	0.561629
2nd Best Set	1.72232	5.48933	1.33691
3rd Best Set	1.76517	3.29319	0.682181
4th Best Set	0.913269	1.81384	1.1494
5th Best Set	0.869327	1.19567	0.572581
6th Best Set	4.13772	0.83092	0.882593
7th Best Set	0.906496	2.89204	2.55669
8th Best Set	1.23329	0.812196	0.921124
9th Best Set	1.14653	1.7763	2.5914
10th Best Set	3.59796	3.89682	0.89399

Table 8. Posterior input parameters: 'best set' ranges found during calibration. REGRESSION VARIABLES.

	Normal to HPV DNA			
	**Low-risk (LR) HPV**	**High-risk 16 (HR-16) HPV**	**High-risk 18 (HR-18) HPV**	**High-risk other (HR-other) HPV**
Best Set	7.14689	4.17272	5.1117	6.10475
2nd Best Set	5.56901	2.86947	6.46357	2.8967
3rd Best Set	6.41645	7.62985	4.151	5.77961
4th Best Set	5.15918	7.07055	6.8038	6.17582
5th Best Set	7.54983	7.11569	6.07369	3.26525
6th Best Set	5.22428	4.6352	6.27671	3.02299
7th Best Set	7.20411	6.35292	5.86792	4.18585
8th Best Set	5.68798	4.84015	3.28093	6.17032
9th Best Set	5.77713	3.80677	5.10114	2.61446
10th Best Set	7.14689	4.17272	5.1117	6.10475
	**CIN 1 to Normal**			
	**Low-risk (LR) HPV**	**High-risk 16 (HR-16) HPV**	**High-risk 18 (HR-18) HPV**	**High-risk other (HR-other) HPV**
Best Set	1.8224	1.68284	4.45645	3.18357
2nd Best Set	2.93868	3.18721	2.45042	4.69422
3rd Best Set	5.58689	2.38326	3.07091	3.17222
4th Best Set	1.43916	5.69735	4.88292	1.24502
5th Best Set	2.00412	4.54546	2.74471	3.57842
6th Best Set	3.34265	0.988405	5.60681	5.38509
7th Best Set	2.56712	3.51813	4.51758	5.12954
8th Best Set	5.56624	0.719508	2.13906	2.51471
9th Best Set	5.18214	4.13439	3.88044	2.33313
10th Best Set	1.8224	1.68284	4.45645	3.18357

Table 8. Posterior input parameters: 'best set' ranges found during calibration. REGRESSION VARIABLES (cont.).

	CIN 2,3 to Normal (70% of women)			
	**Low-risk (LR) HPV**	**High-risk 16 (HR-16) HPV**	**High-risk 18 (HR-18) HPV**	**High-risk other (HR-other) HPV**
Best Set	1.89927	5.09447	1.3789	1.58686
2nd Best Set	1.85223	0.523885	4.62389	5.48724
3rd Best Set	0.536962	3.17252	3.42388	5.73758
4th Best Set	2.88272	2.03862	2.70929	3.96023
5th Best Set	2.60786	1.3153	1.6977	1.53637
6th Best Set	3.90506	4.62244	0.506662	3.12399
7th Best Set	4.47858	2.10466	4.5745	5.62927
8th Best Set	3.6631	5.84419	3.83378	3.12419
9th Best Set	3.20424	0.534472	3.1771	3.92625
10th Best Set	1.89927	5.09447	1.3789	1.58686
	**CIN 2,3 to HPV DNA (15% of women)**			
	**Low-risk (LR) HPV**	**High-risk 16 (HR-16) HPV**	**High-risk 18 (HR-18) HPV**	**High-risk other (HR-other) HPV**
Best Set	1.89927	5.09447	1.3789	1.58686
2nd Best Set	1.85223	0.523885	4.62389	5.48724
3rd Best Set	0.536962	3.17252	3.42388	5.73758
4th Best Set	2.88272	2.03862	2.70929	3.96023
5th Best Set	2.60786	1.3153	1.6977	1.53637
6th Best Set	3.90506	4.62244	0.506662	3.12399
7th Best Set	4.47858	2.10466	4.5745	5.62927
8th Best Set	3.6631	5.84419	3.83378	3.12419
9th Best Set	3.20424	0.534472	3.1771	3.92625
10th Best Set	1.89927	5.09447	1.3789	1.58686

Table 9. Posterior input parameters: 'best set' ranges found during calibration. OTHER VARIABLES.

	Natural Immunity		
	**High-risk 16 (HR-16) HPV**	**High-risk 18 (HR-18) HPV**	**High-risk other (HR-other) HPV**
Best Set	0.991219	0.991219	0.991219
2nd Best Set	0.979688	0.979688	0.979688
3rd Best Set	0.954624	0.954624	0.954624
4th Best Set	0.971936	0.971936	0.971936
5th Best Set	0.997792	0.997792	0.997792
6th Best Set	0.965925	0.965925	0.965925
7th Best Set	0.969257	0.969257	0.969257
8th Best Set	0.999137	0.999137	0.999137
9th Best Set	0.983026	0.983026	0.983026
10th Best Set	0.991219	0.991219	0.991219

Part III: Cervical cancer prevention strategies

Selected model parameters and assumptions for screening and vaccination strategies are shown in **Table 10** (Goldie 2005, Denny 2000 & 2006, Villa 2006).

Table 10. Selected model parameters and assumptions for the performance of screening and vaccination strategies [a].

VARIABLE	*BASE CASE*	*RANGE*
**Population-level variables**		
Vaccine properties [b]		
Age of vaccination	9	
Primary vaccination coverage	70%	0-100%
Percentage of vaccinated girls completing three doses	100%	
Efficacy against infection with HPV 16 and 18	100%	0-100%
Percentage of vaccinated girls with lifelong immunity	100%	0-100%
Waning	None	After 10, 15 years
Discount rate	3%	0%-5%
Cancer costs	1x	0x-2x
Screening properties		
Age at which screening begins	35	40, 45
Primary screening coverage	70%	0-100%
**Performance characteristics of diagnostics [c]**		
Cytology performance for detection of CIN [d]		
Probability of abnormal cytology result given CIN 1	70%	50-90%
Probability of abnormal cytology result given CIN 2,3 or worse	80%	55-93%
Probability of normal cytology result given no CIN	95%	88-97%
Visual inspection with acetic acid performance		
Probability of VIA test positive result given CIN 1	76%	40-81%
Probability of VIA test positive result given CIN 2,3 or worse	76%	40-81%
Probability of VIA test negative result given no CIN (specificity)	81%	40-90%
HPV-DNA testing performance for detection of CIN [e]		
Sensitivity (CIN 2,3+)	83%	70-85%
Specificity	93%	79-94%
**Screening outcomes**		
Ineligible for cryosurgery (%) [f]		
Normal	5%	0-50%
HPV	5%	0-50%
CIN 1	15%	0-50%
CIN 2,3	25%	0-50%
Invasive cancer	90%	50-100%
Loss to follow-up (per visit) [g]	15%	0-50%

Table 10. Selected model parameters and assumptions for the performance of screening and vaccination strategies [a] (cont.).

VARIABLE	*BASE CASE*	*RANGE*
Distribution of treatment methods [h]		
CIN1		
LEEP	33%	
Cryosurgery	67%	
CIN2/3		
LEEP	50%	
Cold knife conization	30%	
Hysterectomy	20%	
**Treatment outcomes and complications** [i]		
Efficacy of treatment		
Short-term efficacy for CIN	100%	50-100%
Persistent HPV infection after treatment	30%	0-100%
Major complications	1%	0-3%
Minor complications	5%	0-15%

HR: High Risk; HPV: human papillomavirus; DNA: deoxyribonucleic acid; CIN: cervical intraepithelial neoplasia; VIA: visual inspection with acetic acid; LEEP: loop electrosurgical excision procedure

a Parameters shown represent the values used in the base case. Sensitivity analyses were conducted by varying each parameter over the range of values shown.

b Vaccine strategies assumed that three doses are given to girls before age 12, and the vaccination series is completed before sexual debut; in sensitivity analyses vaccine efficacy and coverage were varied widely, and alternative assumptions regarding lifelong immunity were assessed.

c Tests for diagnosis included DNA testing for HPV in cervical cell samples with the use of the hybrid-capture method (Hybrid Capture II HPV DNA test, HCII) or rapid HPV test, cytologic examination of cervical cells on a Papanicolaou smear, and visual inspection of the cervix with acetic acid (hereafter referred to as visual inspection). We assumed that screening was performed at a primary-level facility, and included the following strategies in the base case:

Three-visit strategies included an initial screening test in the first visit, colposcopy and biopsy in the case of positive results in a second visit, and treatment of CIN in a third visit.

Two-visit strategies consisted of initial screening followed by treatment, without colposcopic evaluation, of all women with positive screening results.

One-visit strategies incorporated same-day screening and treatment for women with positive screening results.

A single lifetime screen was targeted to 35- or 40-year-old women. The first screening test for 2- and 3-times in a lifetime strategies was targeted to 35-year-old women, with additional screenings conducted at five-year intervals.

d Abnormal cytology was defined as low-grade or high-grade squamous intraepithelial lesions (LSIL or HSIL) for the base case analysis. In sensitivity analyses we assessed the implications of using a more stringent definition, such as HSIL and above.

e Probability of high-risk HPV-DNA positivity on an HPV-DNA test given the presence of high-risk HPV-DNA is assumed to be 100%, however we define the clinically relevant sensitivity of HPV-DNA testing to be the probability of high-risk HPV DNA positivity given CIN 1 and CIN 2, 3+.

f *One-visit and two-visit strategies* involved the use of visual inspection to determine whether women with positive results at screening were eligible for cryosurgery; those with lesions that covered more than 75% of the cervix or that extended to the vaginal wall or 2 mm beyond the tip of the probe used for cryosurgery, and those with anatomical abnormalities of the cervix, were ineligible (Goldie 2005). The value shown is the proportion of women in each underlying disease category who would be ineligible for cryosurgery on the basis of visual inspection of the cervix and would be referred to a secondary-level facility (e.g., a district or regional hospital) for diagnostic testing (e.g., colposcopy and biopsy) and, if necessary, treatment of precancerous lesions with a loop electrosurgical excision procedure, cold-knife conization, or simple hysterectomy, depending on the size and type of lesion. *For three-visit strategies*, women with positive screening results were referred for diagnostic testing, with those who required treatment returning for a third visit. Women in whom cancer was detected were referred to secondary or tertiary care hospitals.

g Loss to follow-up was assumed to occur at each clinical contact. For example, within any particular strategy, a woman who requires three clinical contacts due to an abnormal test result, requirement for further diagnostic testing, and/or any necessary treatment will have approximately a 45% chance of never receiving that care. We defined loss to follow-up as a woman's compliance to return for her screening test results (applicable only to women in two- or three-visit strategies), a woman's compliance to return for diagnostic work-up (applicable only to women ineligible for cryotherapy, and to all women in three-visit strategies), and a woman's compliance to return for treatment (for women ineligible for cryotherapy, and for all women in three-visit strategies).

h For women who were ineligible for cryosurgery, among those with CIN 1, one third would undergo LEEP, and two thirds would undergo cryosurgery; among those with CIN 2 or 3, 50% would undergo LEEP, 30% cold-knife conization and 20% simple hysterectomy.

i Following treatment, women with true CIN have lesions removed with 100% probability. Of the women with true HPV or true CIN that were treated, 30% retain their HPV infection even though lesions had been removed.

Part IV: Additional information about costs

Overview

Since the HPV vaccine price in India is not yet known, nor are the country-specific programmatic costs to deliver a vaccine to a young adolescent age group, we use a composite value defined as the ‘cost per vaccinated girl’, which we assume includes the vaccine cost per dose multiplied by the three required doses, wastage, freight and supplies, administration, immunization support and programmatic costs (Acharya 2002, Kou 2002, Walker 2004, Wolfson 2008, WHO CHOICE). As described later in this section, we distinguish costs dependent on vaccine price (e.g., vaccine wastage, insurance and security fees associated with freight into the country) from those that would be less dependent (e.g., supplies, administration, vaccine support and monitoring/programmatic expenses). We do not include the incremental costs of scaling up vaccination that might be expected after certain thresholds of coverage are attained (e.g., 70%), although we explore a wide range of incremental costs associated with initiating a new program.

We include costs associated with screening, diagnosis and treatment, and categorize these into direct medical costs (e.g., staff, supplies, equipment, and specimen transport), women’s time costs (time spent travelling, waiting, and receiving care), transportation costs, and programmatic costs. Time estimates for various clinical services, for the number and type of follow-up visits, and for hospitalization days are based on previously-published assumptions (Goldie 2005, Goldie 2007); the reader may find additional details in the technical appendices to those previously published analyses.

Selected screening and treatment cost estimates are based on data from a previously published analysis of screening in India (Goldie 2005). Costs were originally reported in 2000 International dollars to facilitate cross-country comparisons. These costs were originally derived in local currency units and costs from other years were converted to local currency units using year-specific exchange rates, adjusted for inflation using country-specific inflation rates, and then converted from local currency units to 2005 International dollars using PPP exchange rates for this analysis (World Bank WDI).

Screening, diagnostic, treatment and cancer costs

Screening, diagnosis and treatment costs are categorized into direct medical costs (e.g., staff, disposable supplies, equipment, specimen transport, facilities, laboratory, hospitalization, and follow-up visits as appropriate for the service), women’s time costs (time spent traveling, waiting, and receiving care), transportation costs, and programmatic costs.

Costs for screening, diagnosis, and pre-cancer treatment, total costs for invasive cervical cancer and patient time and transportation costs are presented in Table 1 of the article. Screening costs include the cost of the office visit, the cost of the test and laboratory processing of the screening sample, and the cost of patient’s transport and time traveling, waiting, and receiving care. Diagnostic costs include the office visit cost, the cost of performing colposcopy, the cost of biopsy and laboratory processing, and the cost of the patient’s time and transport. Costs of pre-cancer

treatment include the facility visit, the cost of the procedure, which include pharmaceuticals and supplies, complications, and hospitalization, as well as the cost of transport and time of the patient. Invasive cancer costs include both direct medical and direct non-medical costs. Direct medical costs of cancer care include staging of cancer severity, hospitalization, stage-appropriate treatment, follow-up visits, as well as patient time and transport costs, which account for approximately 20% of the cancer care.

Direct non-medical costs and time costs associated with cancer care include all patient time in transport, waiting, receiving treatment, and in hospitalization as well as actual transport costs. Time estimates for waiting to receive clinical services include follow-up visits, and hospitalization days, and the costs of two-way transportation from home to the site of care.

Invasive cervical cancer stages 1a1, 1a2, 1b1, 1b2, and 2a are classified as local cancer, stages 2b, 3a, and 3b as regional cancer, and stages 4a and 4b as distant cancer, based on the Federation Internationale de Gynecologie et Obstetriques (FIGO) staging system.

Further details about these methods are provided by Goldie et al. (2005) and Goldie et al. (2007).

Vaccine costs

The composite value, ‘cost per vaccinated girl’, contains the following components:

Table 11. Components of the 'cost per vaccinated girl'

Component	Directly depends on vaccine price
Vaccine dose (three doses)	yes
Vaccine wastage	yes
Immunization supplies (syringes etc.)	no
Supplies wastage	no
Freight into the country	yes (security fees)
Administration charges	no
Vaccine support (cold chain, injection safety and operational costs such as delivery within the country)	no
Monitoring and programmatic services (incremental costs for implementing a young adolescent vaccination program)	no

Categories directly dependent on vaccine price include vaccine wastage and freight into the country (since this component also included insurance and security, which tend to increase as costs of items shipped increase). These costs are considered tradable goods and carry an international dollar price independent of the country setting. These costs are converted to and from Local

Currency Units (LCU) using U.S. dollar direct exchange rates, since by definition, for tradable goods, 1 International Dollar equals 1 U.S. dollar.

Categories less dependent on vaccine price include supplies and supplies wastage (although supply wastage does depend on the supply price), administration, vaccine support and monitoring/programmatic expenses. Categories such as administration, support and programmatic components, considered non-tradable inputs (mostly salaries), tend to vary with the level of development (i.e., GDP) of a country and, as relative salaries increased, so do the costs for these inputs when expressed in International dollars. These costs are converted to and from Local Currency Units using Purchasing Power Parity (PPP) conversion rates.

Vaccine wastage

Wastage rate in different countries, and within countries, is variable, and for this vaccine is uncertain. Wastage rates depend on the specific setting, mode of delivery, and whether the HPV vaccine will continue to be available only in single-dose vials. Even low wastage rates may represent a substantial cost at higher prices (WHO Immunization Financing). Although wastage is reported to be lower with single-dose vials, these are generally more expensive than multi-dose vials. Vials with 10 or more doses tend to be cheaper, but are substantially more troublesome for managing wastage in routine delivery settings. Based on primary data from the Bavi district in rural North Vietnam, wastage rates were found to vary from 10.6% to 32.3% depending on the vaccine (Minh 2008). For the base case, we assume an average of 10-15% wastage, calculated as [(vaccine doses supplied – vaccine doses used)/ vaccine doses supplied]. As there are no data for a new adolescent vaccine in single-dose vials, we vary wastage rates from 5% to 25%.

Vaccine supplies and supply wastage

Costs for disposable items (e.g. syringes, safety boxes) are based on international prices. The primary cost driver in this category is single-use auto-disable (AD) syringes. We use the UNICEF negotiated price of $0.057 per syringe (UNICEF) assuming wastage of 10%.

Freight into country

Our estimates of freight into a country, including insurance, are approximated based on WHO estimates of 6% of the price for vaccines and 15% for vaccine supplies (Kou 2002, WHO Immunization Financing).

Administrative charges

Administrative costs are often reported as a percentage of total immunization program costs, or percentage of costs of a particular program studied. In absence of high quality country-specific data for India, for the purposes of these analyses, administration for vaccination program is divided into 3 costs, low, medium and high, at $0.50, $1.50, and $3. In the base case, we assign categories based on the per dose cost of the vaccine; however, we also vary this assumption in sensitivity analysis to allow for the potential for high administration costs with low vaccine prices and low

administration costs with high vaccine prices. Of note, the potential additional costs associated with a new adolescent vaccine schedule are explored by varying the category of costs, vaccine support.

<u>Vaccine support</u>

These costs are also highly variable within countries, and are typically reported as a percentage of total vaccination/immunization program costs.

Cold chain, injection safety and operational costs such as delivery within the country

For our analyses, for all costs per fully-immunized girl (FIG) over $10, we assume that cold chain, injection safety and operational costs together as a category would account for $2.94. As a percentage of total cost, this ranges from almost 30% ($10/FIG) to less than 10% ($300/FIG). For costs below $10/FIG, we assume that this value is reduced to $2/FIG, which accounts for roughly the same percentage range (10%-30%).

Monitoring and programmatic services

These included an exploration of the additional incremental costs for implementing a young adolescent vaccination program. While there have been studies to assess costs associated with childhood immunization programs (Khan 1998, Kaddar 1999, Levin 2001; Miller 2000, Walker 2004, Waters 2004), the financial requirements necessary for social mobilization and an education campaign for a new vaccine, particularly one that targets young adolescent girls, are not known. Wolfson et al. (2008) estimated that the per person targeted (between ages nine months and 29 years) costs for meningococcal vaccines ranged between US$0.17-1.53 and campaigns targeting women of childbearing age to reduce maternal and neonatal tetanus ranged from US$0.19-1.51. As such, for per dose costs (US$) of $0.55, $2, and $5 (corresponding to a cost per immunized girl of I$5, I$10 and I$25), we assign values ranging from $0 to $2 for the incremental per person targeted costs of a new program. The costs associated with social outreach reported for selected childhood programs ranged from 3.4% (Morocco) to 15% (Ghana) (Khan 1998, Kaddar 1999, Levin 2001). Assuming an additional outreach cost of $2/FIG, this translates to 20% at $10/FIG and 2% at $100/FIG.

A stylized example of the components of the composite value, as it varied from I$10 to I$75, is shown in the figure below. For example, for a composite cost of I$50 per vaccinated girl, we assume three doses of vaccine at (US$) $12.25 each; wastage of $5.51; freight and supplies of $1.30; administration of $1.50; immunization support costs of $2.94; and the incremental costs of a new adolescent program, $2. Of note, we vary the *total composite cost* in the model, and therefore, by providing results on such a wide range of *total composite values*, we permit the reader to elucidate two kinds of insights from the results: the effect of the *total cost per immunized girl* on the cost-effectiveness of HPV 16,18 vaccination, and the effect of varying a *specific component* of the total cost.

Figure 4. Cost per vaccinated girl

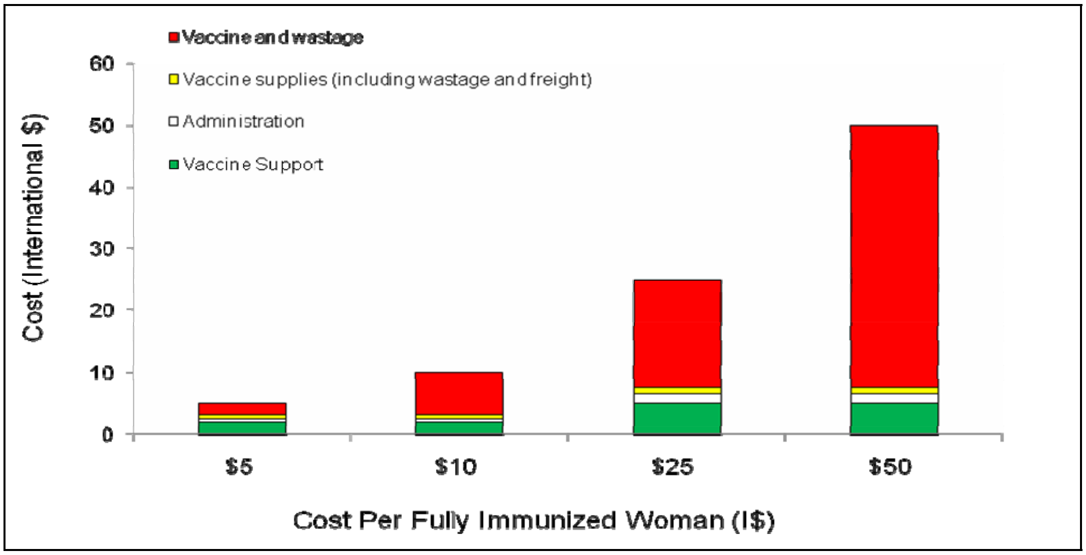

Estimating financial costs to facilitate face validity exercises and to assess affordability

For the main cost-effectiveness analysis, costs are presented in 2005 international dollars, a currency that provides a means of translating and comparing costs among countries, taking into account differences in purchasing power. The choice of using international dollars for the cost-effectiveness analysis allows for broad comparison across regions. In contrast, projections of financial resources to assess local and regional affordability are presented also in U.S. dollars. Often financial costs associated with short-term payments required for a program are of interest to local payers as they assess their immediate budgets and fiscal space. For the latter objective, we translated selected results that rely on international dollars to local currency units or U.S. dollars in a particular country setting. An example of the conversion process used to present these costs in additional currencies for local and regional decision makers is shown below.

Figure 5. Currency conversions

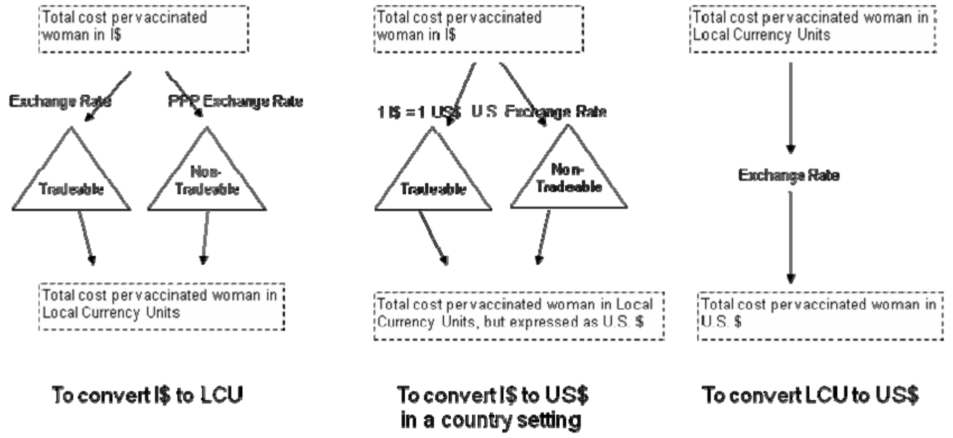

Tradeable goods include: The vaccine price, wastage & supplies. Non-tradeable goods include: administration & vaccine support categories, including programmatic and operational costs.

Example of a cost conversion

For India, we use an exchange rate (Local Currency Units per U.S. Dollar) of 44.10 and a Purchasing Power Parity exchange rate (Local Currency Units per International Dollar) of 9.44 (World Bank WDI).

Table 12. Stylized example of conversion from international dollars to local currency

	International $	Tradable/ Non-Tradable	Local Currency Unit	U.S. $ equivalent
	**$25**		**879.29**	**18.71**
Vaccine cost	15	Tradable	661.50	15.00
Vaccine wastage	2.25	Tradable	99.22	2.25
Vaccine supplies (including wastage & freight)	1.31	Tradable	57.77	1.31
Administration	1.5	Non-Tradable	14.16	0.03
Vaccine Support				
Monitoring & Programmatic services	2	Non-Tradable	18.88	0.05
Cold chain, injection safety, operational costs	2.94	Non-Tradable	27.75	0.07

The U.S. dollar equivalent can be calculated in two ways. The method demonstrated above expresses the international dollar price as U.S. dollars in a country setting, where tradable goods were converted using the assumption that 1 U.S.$ = 1 I$, and non-tradable goods are converted using the LCU to U.S. dollar exchange rate. Alternatively, conversion to local currency units can be

accomplished first and then exchanged directly to U.S. dollars using only exchange rates. For India, that results in an equivalent price of US$19.94.

Further sources of information and discussion on the issues of valuing costs in developing countries, of traded versus non-traded goods and how to handle each of them, how to transfer costs across time and location as well as how and when to present results in International dollars can be found in Hutton and Baltussen (2005), Johns et al. (2003) and Tan-Torres Edejer et al. (2003).

Face validity exercises

Three studies that reported expenditures for childhood vaccination programs in developing countries (Khan 1998, Kaddar 1999, Levin 2001), showed costs per fully-immunized child to range from $11.76 (Bangladesh) to $21.00 (Morocco). All 3 of these countries had a per dose price for the EPI vaccine of less than $1, which translated to per immunized child costs of over $10. More recently, the WHO reported cost per DPT-3 immunized child ranging from $4.10 in Ghana to $9.60 in Mozambique, again with vaccines with a per dose price less than $1. Using the stylized methods outlined above, we would assume a cost per vaccinated girl of I$6.51 when the vaccine cost per dose is at US$1 (or I$8.51 if social outreach is included).

Part V: Additional results

Additional results are shown for strategies utilizing VIA and Rapid HPV test. We varied the following variables: vaccination cost, I$20 and I$50; VIA sensitivity, 40%-70%, HPV DNA test cost, I$1.30-I$10 (with hybrid capture method), and Rapid HPV test sensitivity and specificity, 70%-100%. In the base case for VIA and Rapid HPV, we assumed VIA sensitivity of 76% and specificity of 81% with a cost of I$1.30, and a sensitivity of 90% and specificity of 84% for Rapid HPV test.

Table 13. Sensitivity analysis for VIA varying VIA sensitivity, and VIA and vaccine costs [a, b].

STRATEGIES		VACCINE COSTS I$20 *Approximate per-dose cost (I$4)*					VACCINE COSTS I$50 *Approximate per-dose cost (I$12)*				
		VIA Sensitivity									
		**0.4**	**0.5**	**0.6**	**0.7**	**0.81**	**0.4**	**0.5**	**0.6**	**0.7**	**0.81**
		VIA Costs I$1.30									
Vaccination		**I$255**	**I$285**	**I$355**	dom[c]		dom[c]	dom[c]	dom[c]	dom[c]	
Screening 3 times per lifetime at age 35, 40 & 45	1-visit VIA	dom[c]	**I$210**	**I$140**	**I$95**		**I$345**	**I$210**	**I$145**	**I$100**	
	2-visit HPV	dom[c]	dom[c]	dom[c]	dom[c]		**I$710**	**I$860**	**I$1,065**	**I$1,360**	
Vaccination + Screening 3 times per lifetime at age 35, 40 & 45	1-visit VIA	dom[c]	**I$825**	**I$535**	**I$430**		dom[c]	dom[c]	dom[c]	**I$1,485**	
	2-visit HPV	**I$1,090**	**I$1,175**	**I$1,390**	**I$1,680**		**I$1,575**	**I$1,575**	**I$1,575**	**I$1,680**	
		VIA Costs I$2.00									
Vaccination		**I$255**	**I$255**	**I$316**	**I$405**	dom[c]	dom[c]	dom[c]	dom[c]	dom[c]	dom[c]
Screening 3 times per lifetime at age 35, 40 & 45	1-visit VIA	dom[c]	dom[c]	**I$181**	**I$130**	**I$110**	**I$420**	**I$265**	**I$185**	**I$135**	**I$115**
	2-visit HPV	dom[c]	dom[c]	dom[c]	dom[c]	dom[c]	**I$680**	**I$825**	**I$1,020**	**I$1,310**	dom[c]
Vaccination + Screening 3 times per lifetime at age 35, 40 & 45	1-visit VIA	dom[c]	**I$980**	**I$635**	**I$470**	**I$445**	dom[c]	dom[c]	dom[c]	**I$1,545**	**I$1,465**
	2-visit HPV	**I$1,090**	**I$1,125**	**I$1,335**	**I$1,620**	**I$1,815**	**I$1,575**	**I$1,575**	**I$1,575**	**I$1,615**	**I$1,815**

Table 13. Sensitivity analysis for VIA varying VIA sensitivity, and VIA and vaccine costs [a, b]. (cont.).

STRATEGIES		VACCINE COSTS I$20 *Approximate per-dose cost (I$4)*					VACCINE COSTS I$50 *Approximate per-dose cost (I$12)*				
		VIA Sensitivity									
		0.4	0.5	0.6	0.7	0.81	0.4	0.5	0.6	0.7	0.81
		VIA Costs I$5.00									
Vaccination		**I$255**	**I$255**	**I$255**	dom[c]	**I$280**	dom[c]	dom[c]	dom[c]	dom[c]	dom[c]
Screening 3 times per lifetime at age 35, 40 & 45	1-visit VIA	dom[c]	dom[c]	dom[c]	dom[c]	**I$240**	dom[c]	I$480	I$355	I$275	I$240
	2-visit HPV	dom[c]	dom[c]	dom[c]	dom[c]	dom[c]	**I$605**	**I$685**	**I$850**	**I$1,095**	**I$1,290**
Vaccination + Screening 3 times per lifetime at age 35, 40 & 45	1-visit VIA	dom[c]	dom[c]	**I$1,040**	**I$780**	**I$680**	dom[c]	dom[c]	dom[c]	dom[c]	dom[c]
	2-visit HPV	**I$1,090**	**I$1,090**	**I$1,115**	**I$1,355**	**I$1,520**	**I$1,575**	**I$1,575**	**I$1,575**	**I$1,575**	**I$1,575**
		VIA Costs I$7.00									
Vaccination		**I$255**	**I$255**	**I$255**	**I$255**	**I$255**	dom[c]	dom[c]	dom[c]	dom[c]	dom[c]
Screening 3 times per lifetime at age 35, 40 & 45	1-visit VIA	dom[c]	dom[c]	dom[c]	dom[c]	dom[c]	dom[c]	dom[c]	**I$470**	**I$370**	**I$330**
	2-visit HPV	dom[c]	dom[c]	dom[c]	dom[c]	dom[c]	**I$605**	**I$605**	**I$740**	**I$955**	**I$1,125**
Vaccination + Screening 3 times per lifetime at age 35, 40 & 45	1-visit VIA	dom[c]	dom[c]	dom[c]	**I$980**	**I$864**	dom[c]	dom[c]	dom[c]	dom[c]	dom[c]
	2-visit HPV	**I$1,090**	**I$1,090**	**I$1,090**	**I$1,180**	**I$1,325**	**I$1,575**	**I$1,575**	**I$1,575**	**I$1,575**	**I$1,575**
		VIA Costs I$10.00									
Vaccination		**I$255**	**I$255**	**I$255**	**I$255**	**I$255**	dom[c]	dom[c]	dom[c]	dom[c]	dom[c]
Screening 3 times per lifetime at age 35, 40 & 45	1-visit VIA	dom[c]	dom[c]	dom[c]	dom[c]	dom[c]	dom[c]	dom[c]	dom[c]	**I$511**	**I$458**
	2-visit HPV	dom[c]	dom[c]	dom[c]	dom[c]	dom[c]	**I$603**	**I$603**	**I$603**	**I$741**	**I$877**
Vaccination + Screening 3 times per lifetime at age 35, 40 & 45	1-visit VIA	dom[c]	dom[c]	dom[c]	dom[c]	dom[c]	dom[c]	dom[c]	dom[c]	dom[c]	dom[c]
	2-visit HPV	**I$1,088**	**I$1,088**	**I$1,088**	**I$1,088**	**I$1,088**	**I$1,575**	**I$1,575**	**I$1,575**	**I$1,575**	**I$1,575**

[a] After eliminating strategies that were dominated, incremental cost-effectiveness ratios were calculated for the remaining strategies. The incremental cost-effectiveness ratios shown represent the mean costs divided by the mean effects of a sample of good-fitting parameter sets.

[b] The cost per vaccinated girl included three doses of vaccine, wastage, freight and supplies, administration, and immunization support and programmatic costs.

[c] dom: These strategies were either more costly and less effective, or more costly and less cost-effective, than alternative options, and were thus considered dominated.

HPV: Human Papillomavirus

VIA: Visual Inspection with Acetic Acid

Table 14. Sensitivity analysis for rapid HPV test varying test sensitivity and specificity, and rapid HPV and vaccine costs [a, b].

STRATEGIES		HPV (hybrid capture method) and rapid HPV Cost I$10.30									
		VACCINE COSTS I$20 *Approximate per-dose cost (I$4)*					VACCINE COSTS I$50 *Approximate per-dose cost (I$12)*				
		Rapid HPV Specificity									
		0.7	0.8	0.84	0.9	1.0	0.7	0.8	0.84	0.9	1.0
		Rapid HPV Sensitivity 0.7									
Vaccination		**I$255**		**I$255**			dom[c]		dom[c]		
Screening 3 times per lifetime at age 35, 40 & 45	1-visit Rapid HPV	dom[c]		dom[c]			**I$418**		**I$355**		
	2-visit HPV	dom[c]		dom[c]			dom[c]		dom[c]		
Vaccination + Screening 3 times per lifetime at age 35, 40 & 45	1-visit Rapid HPV	**I$788**		**I$678**			**I$1,455**		**I$1,452**		
	2-visit HPV	**I$2,990**		**I$3,685**			**I$2,990**		**I$3,685**		
		Rapid HPV Sensitivity 0.8									
Vaccination				**I$255**					dom[c]		
Screening 3 times per lifetime at age 35, 40 & 45	1-visit Rapid HPV			dom[c]					**I$309**		
	2-visit HPV			dom[c]					dom[c]		
Vaccination + Screening 3 times per lifetime at age 35, 40 & 45	1-visit Rapid HPV			**I$601**					**I$1,535**		
	2-visit HPV			**I$12,803**					**I$12,803**		
		Rapid HPV Sensitivity 0.9									
Vaccination		**I$255**	**I$255**	**I$255**	**I$347**	dom[c]	dom[c]	dom[c]	dom[c]	dom[c]	dom[c]
Screening 3 times per lifetime at age 35, 40 & 45	1-visit Rapid HPV	dom[c]	dom[c]	dom[c]	**I$251**	**I$214**	**I$326**	**I$289**	**I$274**	**I$252**	**I$215**
	2-visit HPV	dom[c]	dom[c]	dom[c]	dom[c]	dom[c]	dom[c]	dom[c]	dom[c]	dom[c]	dom[c]
Vaccination + Screening 3 times per lifetime at age 35, 40 & 45	1-visit Rapid HPV	**I$633**	**I$568**	**I$542**	**I$502**	**I$489**	**I$1,626**	**I$1,622**	**I$1,620**	**I$1,618**	**I$1,618**
	2-visit HPV	dom[c]	dom[c]	dom[c]	dom[c]	dom[c]	dom[c]	dom[c]	dom[c]	dom[c]	dom[c]
		Rapid HPV Sensitivity 1.0									
Vaccination				dom[c]		dom[c]			dom[c]		dom[c]
Screening 3 times per lifetime at age 35, 40 & 45	1-visit Rapid HPV			**I$246**		**I$174**			**I$247**		**I$175**
	2-visit HPV			dom[c]		dom[c]			dom[c]		dom[c]
Vaccination + Screening 3 times per lifetime at age 35, 40 & 45	1-visit Rapid HPV			**I$521**		**I$615**			**I$1,705**		**I$1,950**
	2-visit HPV			dom[c]		dom[c]			dom[c]		dom[c]

Table 14. Sensitivity analysis for rapid HPV test varying test sensitivity and specificity, and rapid HPV and vaccine costs [a, b] (cont.).

STRATEGIES		HPV (hybrid capture method) Costs I$10.30 and rapid HPV Costs I$5.15									
		VACCINE COSTS I$20 *Approximate per-dose cost (I$4)*					VACCINE COSTS I$50 *Approximate per-dose cost (I$12)*				
		Rapid HPV Specificity									
		0.7	0.8	0.84	0.9	1.0	0.7	0.8	0.84	0.9	1.0
		Rapid HPV Sens 0.7									
Vaccination		**I$285**		dom[c]			dom[c]		dom[c]		
Screening 3 times per lifetime at age 35, 40 & 45	1-visit Rapid HPV	**I$247**		**I$184**			**I$248**		**I$185**		
	2-visit HPV	dom[c]		dom[c]			dom[c]		dom[c]		
Vaccination + Screening 3 times per lifetime at age 35, 40 & 45	1-visit Rapid HPV	**I$505**		**I$433**			**I$1,455**		**I$1,451**		
	2-visit HPV	**I$4,781**		**I$5,477**			**I$4,781**		**I$5,477**		
		Rapid HPV Sens 0.8									
Vaccination				dom[c]			dom[c]	dom[c]	dom[c]	dom[c]	dom[c]
Screening 3 times per lifetime at age 35, 40 & 45	1-visit Rapid HPV			**I$155**			**I$187**	**I$150**	**I$135**	**I$112**	**I$75**
	2-visit HPV			dom[c]			dom[c]	dom[c]	dom[c]	dom[c]	dom[c]
Vaccination + Screening 3 times per lifetime at age 35, 40 & 45	1-visit Rapid HPV			**I$462**			**I$1,625**	**I$1,621**	**I$1,620**	**I$1,618**	**I$1,617**
	2-visit HPV			**I$18,918**			dom[c]	dom[c]	dom[c]	dom[c]	dom[c]
		Rapid HPV Sens 0.9									
Vaccination		dom[c]	dom[c]	dom[c]	dom[c]	dom[c]	dom[c]	dom[c]	dom[c]	dom[c]	dom[c]
Screening 3 times per lifetime at age 35, 40 & 45	1-visit Rapid HPV	**I$186**	**I$149**	**I$134**	**I$111**	**I$74**	**I$326**	**I$289**	**I$274**	**I$252**	**I$215**
	2-visit HPV	dom[c]	dom[c]	dom[c]	dom[c]	dom[c]	dom[c]	dom[c]	dom[c]	dom[c]	dom[c]
Vaccination + Screening 3 times per lifetime at age 35, 40 & 45	1-visit Rapid HPV	**I$496**	**I$493**	**I$492**	**I$490**	**I$488**	**I$1,626**	**I$1,622**	**I$1,620**	**I$1,618**	**I$1,618**
	2-visit HPV	dom[c]	dom[c]	dom[c]	dom[c]	dom[c]	dom[c]	dom[c]	dom[c]	dom[c]	dom[c]
		Rapid HPV Sens 1.0									
Vaccination				dom[c]		dom[c]		dom[c]		dom[c]	dom[c]
Screening 3 times per lifetime at age 35, 40 & 45	1-visit Rapid HPV			**I$117**		**I$50**		**I$118**		**I$51**	**I$175**
	2-visit HPV			dom[c]		dom[c]		dom[c]		dom[c]	dom[c]
Vaccination + Screening 3 times per lifetime at age 35, 40 & 45	1-visit Rapid HPV			**I$521**		**I$615**		**I$1,705**		**I$1,950**	**I$1,950**
	2-visit HPV			dom[c]		dom[c]		dom[c]		dom[c]	dom[c]

[a] After eliminating strategies that were dominated, incremental cost-effectiveness ratios were calculated for the remaining strategies. The incremental cost-effectiveness ratios shown represent the mean costs divided by the mean effects of a sample of good-fitting parameter sets.

[b] The cost per vaccinated girl included three doses of vaccine, wastage, freight and supplies, administration, and immunization support and programmatic costs.

[c] dom: These strategies were either more costly and less effective, or more costly and less cost-effective, than alternative options, and were thus considered dominated.

References

Acharya A, Diaz-Ortega JL, Tambini G, de Quadros C, Arita I. Cost-effectiveness of measles elimination in Latin America and the Caribbean: a prospective analysis. Vaccine. 2002 Sep 10;20(27-28):3332-41.

Bhatla N, Dar L, Patro AR, Kriplani A, Gulati A, Verma K, Broor S, Shah KV, Gravitt PE. Human papillomavirus type distribution in cervical cancer in Delhi, India. Int J Gynecol Pathol 2006;25(4):398-402

Denny L, Kuhn L, Pollack A, Wainwright H, Wright TC Jr. Evaluation of alternative methods of cervical cancer screening for resource-poor settings. Cancer 2000;89: 826-33.

Denny L, Quinn M, Sankaranarayanan R. Chapter 8: Screening for cervical cancer in developing countries. Vaccine*.* 2006;24 (Suppl 3):S71-S77.

Franceschi S, Rajkumar T, Vaccarella S, Gajalakshmi V, Sharmila A, Snijders PJ, Munoz N, Meijer CJ, Herrero R. Human papillomavirus and risk factors for cervical cancer in Chennai, India: a case-control study. Int J Cancer 2003;107(1):127-33

Franceschi S, Rajkumar R, Snijders PJ, Arslan A, Mahe C, Plummer M, Sankaranarayanan R, Cherian J, Meijer CJ, Weiderpass E. Papillomavirus infection in rural women in southern India. Br J Cancer 2005;92(3):601-6.

Goldie SJ, Gaffikin L, Goldhaber-Fiebert JD, Gordillo-Tobar A, Levin C, Mahe C, Wright TC; Alliance for Cervical Cancer Prevention Cost Working Group. Cost-effectiveness of cervical cancer screening in five developing countries. N Engl J Med 2005;353(20):2158-68.

Goldie SJ, Kim JJ, Kobus K, Goldhaber-Fiebert JD, Salomon J, O'Shea MKH, Bosch FX, de Sanjose S, Franco EL. Cost-effectiveness of HPV 16, 18 vaccination in Brazil. Vaccine 2007;25:6257-6270.

Hutton G, Baltussen R. Cost valuation in resource-poor settings. Health Policy and Planning. 2005. 20:252-259.

Johns B, Baltussen R, Hutubessy R. Programme costs in the economic evaluation of health interventions. Cost Effectiveness and Resource Allocation 2003; 1 (1).

Kaddar M, Mookherji S, DeRoeck D, Antona D. September 1999. Case Study on the Costs and Financing of Immunization Services in Morocco. Special Initiatives Report No. 18. Bethesda, MD: Partnerships for Health Reform Project, Abt Associates Inc.

Khan MM and Yoder RA. September 1998. Expanded Program on Immunization in Bangladesh: Cost, Cost-Effectiveness, and Financing Estimates. Technical Report No. 24. Bethesda, MD: Partnerships for Health Reform Project, Abt Associates Inc.

Kim JJ, Kuntz KM, Stout NK, Mahmud S, Villa LL, Franco EL, et al. Multi-parameter calibration of a natural history model of cervical cancer. Am J Epidemiol 2007;166(2):137-50

Kou U. Guidelines for estimating costs of introducing vaccines into the national immunization system. Department of Vaccines and Biologicals. Geneva: World Health Organization; 2002.

Available online at http://whqlibdoc.who.int/hq/2002/WHO_V&B_02.11.pdf (Last accessed April 1, 2008)

Laikangbam P, Sengupta S, Bhattacharya P, Duttagupta C, Dhabali Singh T, Verma Y, Roy S, Das R, Mukhopadhyay S. A comparative profile of the prevalence and age distribution of human papillomavirus type 16/18 infections among three states of India with focus on northeast India. Int J Gynecol Cancer 2007;17(1):107-17

Levin A, England S, Jorissen J, Garshong B, Teprey J. September 2001. Case Study on the Costs and Financing of Immunization Services in Ghana. Bethesda, MD: The Partners for Health Reformplus Project, Abt Associates Inc.

Miller MA, McCann L. Policy analysis of the use of hepatitis b, haemophilus influenzae type b, streptococcus pneumoniae-conjugate and rotavirus vaccines in national immunization schedules. Health Economics 2000;9:19-35

Minh HV, Yen NTB, Giang KB, Huong DL, Huong NT, Wright P. Cost of providing the expanded program on immunization: findings from a facility-based study in Viet Nam, 2005. Bull WHO 2008;86(6):417-496.

Munirajan AK, Kannan K, Bhuvarahamurthy V, Ishida I, Fujinaga K, Tsuchida N, Shanmugam G. The status of human papillomavirus and tumor suppressor genes p53 and p16 in carcinomas of uterine cervix from India. Gynecol Oncol 1998;69(3):205-9

New York State Department of Health. About age adjusted rates, 95% confidence intervals and unstable rates. Available online at http://www.health.state.ny.us/statistics/cancer/registry/age.htm. (Last accessed October 2, 2007)

Parkin, D.M., Whelan, S.L., Ferlay, J., and Storm, H. Cancer Incidence in Five Continents, Vol. I to VIII. IARC CancerBase No. 7, Lyon, 2005.

Peedicayil A, Abraham P, Sathish N, John S, Shah K, Sridharan G, Gravitt P. Human papillomavirus genotypes associated with cervical neoplasia in India. Int J Gynecol Cancer 2006;16(4):1591-5

Reiczigel J. Confidence intervals for the binomial parameter: some new considerations. Stat Med 2003;22:611-621.

Sankaranarayanan R, Chatterji R, Shastri SS, Wesley RS, Basu P, Mahe C, Muwonge R, Seigneurin D, Somanathan T, Roy C, Kelkar R, Chinoy R, Dinshaw K, Mandal R, Amin G, Goswami S, Pal S, Patil S, Dhakad N, Frappart L, Fontaniere B. Accuracy of human papillomavirus testing in primary screening of cervical neoplasia: results from a multicenter study in India. Int J Cancer 2004;112(2):341-7

Smith JS, Lindsay L, Hoots B, Keys J, Franceschi S, Winer R, Clifford GM. Human papillomavirus type distribution in invasive cervical cancer and high-grade cervical lesions: a meta-analysis update. Int J Cancer 2007;121,621–632.

Sowjanya AP, Jain M, Poli UR, Padma S, Das M, Shah KV, Rao BN, Devi RR, Gravitt PE, Ramakrishna G. Prevalence and distribution of high-risk human papilloma virus (HPV) types in

invasive squamous cell carcinoma of the cervix and in normal women in Andhra Pradesh, India. BMC Infect Dis 2005;5:116

Stinnett AA, Paltiel DA. Estimating CE ratios under second-order uncertainty: the mean ratio versus the ratio of the means. Med Decis Making 1997;17:483–9.

Tan-Torres Edejer T, Baltussen R, Adam T, Hutubessy R, Acharya A, Evans DB, and Murray CJL, eds. Making Choices in Health: WHO Guide to Cost-Effectiveness Analysis. Geneva, Switzerland: World Health Organization, 2003.

Tobi H, van den Berg PB, de Jong-van den Berg LT. Small proportions: what to report for confidence intervals? Pharmacoepidemiol Drug Saf 2005;14:239-247.

UNICEF Procuring supplies for children. Available online at http://www.unicef.org/supply/ (Last accessed September 2, 2007)

Villa LL, Denny L. Methods for detection of HPV infection and its clinical utility. Int J Gynecol Obstet. 2006;94:S71-80,

Walker D, Mosqueira NR, Penny ME, Lanata CF, Clark AD, Sanderson CF, Fox-Rushby JA. Variation in the costs of delivering routine immunization services in Peru. Bull World Health Organ, 2004;82(9):676-82

Waters HR, Dougherty L, Simon-Pierce T, Tran N, Wysonge CS, Long K, Wolfe ND, Burke DS. Coverage and costs of childhood immunizations in Cameroon. Bulletin of the World Health Organization 2004;82(9):668-75

Wolfson LJ, Gasse F, Lee-Martin SP, Lydon P, Magan A, Tibouti A, Johns B, Hutubessy R, Salama P, Okwo-Bele JM. Estimating the costs of achieving the WHO–UNICEF Global Immunization Vision and Strategy, 2006–2015. Bull World Health Organ 2008;86:27–39.

World Bank World Development Indicators, WDI Online. Available online at http://www.worldbank.org/ (Last accessed January 24, 2008).

World Health Organization (WHO). WHO Immunization Financing. Available online at http://www.who.int/immunization_financing/en/ (Last accessed March 16, 2008)

World Health Organization Statistical Information System: CHOICE (CHOosing Interventions that are Cost Effective). Available online at http://www.who.int/choice/en/. (Last accessed October 17, 2007)
